# Cross-species hepatic transcriptomics identify conserved immune-metabolic reprogramming in acute-on-chronic liver failure progression

**DOI:** 10.3389/fimmu.2026.1702689

**Published:** 2026-02-24

**Authors:** Panyu Chen, Yun Song, Xiao Lin, Zhaokai Zeng, Wenxi Su, Yunyun Ren, Chen Wang, Kang He, Min Shi, Yugang Wang

**Affiliations:** 1 Department of Gastroenterology, Tongren Hospital, Shanghai Jiao Tong University School of Medicine, Shanghai, China; 2 Key Laboratory for Translational Research and Innovative Therapeutics of Gastrointestinal Oncology, Tongren Hospital, Shanghai Jiao Tong University School of Medicine, Shanghai, China; 3 Hongqiao International Institute of Medicine, Tongren Hospital, Shanghai Jiao Tong University School of Medicine, Shanghai, China; 4 State Key Laboratory of Drug Research, Shanghai Institute of Materia Medica, Chinese Academy of Sciences, Shanghai, China; 5 Department of Infectious Disease, Renji Hospital, School of Medicine, Shanghai Jiao Tong University, Shanghai, China; 6 Department of Liver Surgery, Renji Hospital, School of Medicine, Shanghai Jiao Tong University, Shanghai, China; 7 Department of Gastroenterology, Xinhua Hospital, School of Medicine, Shanghai Jiaotong University, Shanghai, China

**Keywords:** acute-on-chronic liver failure, immune dysfunction, liver cirrhosis, metabolic disorder, monocyte/macrophage, transcriptional profiles

## Abstract

**Background:**

Acute-on-chronic liver failure is a fatal syndrome involving sudden hepatic deterioration in patients with chronic liver disease, resulting in high short-term mortality. The intrahepatic molecular mechanisms that drive disease progression are poorly understood, partly due to limited access to human liver tissues.

**Method:**

Transcriptomic profiling of liver tissues from patients with hepatitis B virus-related acute-on-chronic liver failure and a corresponding murine model was performed. Comparative analyses were conducted across disease stages to delineate the dynamic immune and metabolic trajectories.

**Result:**

The analysis uncovered a conserved immune-metabolic dysregulation during disease progression. In both patients and mice, immune activation-characterized by monocyte and macrophage infiltration and altered cytokine signaling-coincided with progressive metabolic failure, including the suppression of mitochondrial functions. The murine model further demonstrated a transition from an early stage of hyperinflammation to a later stage of immune exhaustion. Moreover, several monocyte and macrophage-associated genes were identified as conserved markers that correlate with disease severity, highlighting their potential as biomarkers or therapeutic targets.

**Conclusion:**

This study defines a conserved immune-metabolic interplay during the progression of hepatitis B virus-related acute-on-chronic liver failure and validates the murine model’s accuracy for studying the disease’s terminal stage. The identified dysregulation of immune cells and metabolic pathways presents actionable targets for developing stage-specific therapies intended to disrupt the disease’s vicious immune-metabolic cycle.

## Introduction

Acute-on-Chronic Liver Failure (ACLF) is a life-threatening syndrome marked by acute hepatic deterioration in patients with chronic liver diseases (1–3). Although its etiologies vary by region, its consistently high short-term mortality makes it a major cause of death in cirrhotic and chronic liver disease worldwide (2, 4–6). To date, no effective pharmacological treatment exists; management relies on etiology-specific measures and supportive care (7–9). In many cases, liver transplantation is the only curative option but is limited by donor scarcity and high costs (8, 10, 11). These challenges highlight the urgent need to clarify the mechanisms underlying ACLF progression and identify new therapeutic targets (12–14).

The etiologies and diagnostic criteria for ACLF differ substantially between Eastern and Western populations (15–19). HBV-ACLF was defined by the Chinese Group on the Study of Severe Hepatitis B as a distinct clinical entity, different from the alcohol-associated ACLF characterized in Western populations by the CANONIC study of the Chronic Liver Failure Consortium (19, 20). The CANONIC study highlighted overwhelming systemic inflammation as a key driver of acute decompensation in patients with alcohol- or hepatitis C virus–related ACLF (20). In contrast, transcriptomic analyses of peripheral blood mononuclear cells from HBV-ACLF patients in the Chinese Group on the Study of Severe Hepatitis B cohort revealed that dysregulated immune and metabolic pathways constitute a central axis of disease initiation and progression in this population (21).

Although systemic inflammation, immune dysfunction and metabolism disorder are known contributors to ACLF pathogenesis, the intrahepatic molecular events that drive disease progression remain poorly defined (21–24). Advanced liver disease is also accompanied by severe immune dysfunction, which is associated with the high short-term mortality observed in patients with ACLF (25, 26). This condition is characterized by an early and sustained systemic inflammatory response that intensifies with progressive hepatic injury, eventually culminating in immune exhaustion and impaired pathogen defense (25, 27–29). Referred to as “immune paralysis,” this phenomenon is not unique to advanced chronic liver disease and has also been reported in other conditions of profound immune suppression, such as severe sepsis or septic shock (27, 28, 30–33). However, the pathogenic mechanisms underlying immune dysfunction and immunodeficiency remain incompletely understood (25, 31). Due to the limited availability of human liver samples, most existing studies rely on peripheral blood immune cells or rodent models, which fail to fully capture the liver-localized regulatory networks (21, 34).

Emerging evidence positions metabolic disorder as a central pillar of ACLF pathogenesis, beyond its classical role in chronic liver disease (35, 36). The liver, as the body’s metabolic hub, governs lipid oxidation, gluconeogenesis, detoxification, and methyl donor synthesis-processes critical for maintaining systemic homeostasis (37). In chronic liver disease, persistent insults (e.g., HBV, alcohol) progressively erode metabolic flexibility, priming the liver for acute decompensation (38). Concurrently, systemic inflammation-marked by cytokine storms and myeloid cell infiltration-further disrupts metabolic networks by inhibiting PPAR signaling and exacerbating oxidative stress (21). This creates a vicious cycle: metabolic insufficiency limits ATP-dependent immune effector functions, while inflammatory mediators suppress reparative metabolic pathways (39). Such immune-metabolic crosstalk is increasingly recognized as a hallmark of ACLF progression, yet the liver-intrinsic drivers of this axis remain poorly defined.

To better understand the molecular basis of HBV-ACLF progression, we performed transcriptomic profiling of liver tissues from both patients and mouse models across different disease stages. By integrating human and murine data, we aimed to identify conserved transcriptional programs and critical pathways that orchestrate intrahepatic immune-metabolic dysfunction. Our cross-species analysis provides insights into the conserved mechanisms underlying HBV-ACLF and offers a valuable foundation for therapeutic target discovery.

## Materials and methods

### Patients and study samples

All human participants were enrolled at Renji Hospital, affiliated with Shanghai Jiao Tong University School of Medicine, and provided written informed consent prior to inclusion. The study protocol adhered to the ethical principles outlined in the Declaration of Helsinki and was reviewed and approved by the Institutional Ethics Committee of Renji Hospital (approval numbers KY2020–055 and KY2021-063-B).

Liver specimens were collected from individuals aged 18 years or older who underwent either liver biopsy or liver transplantation at the same institution. In total, 64 human liver tissue samples were included for analysis. Hepatitis B virus (HBV) infection status was determined based on HBV DNA quantification and serological testing for HBV surface and core antigens and antibodies. Liver tissues from healthy controls (HC, n = 19) were obtained from residual biopsy material of donor livers after transplantation. Diagnosis of liver cirrhosis (LC, n = 22) was established through histological evaluation and clinical criteria. Patients classified under ACLF (n = 23) met the diagnostic definitions established by both the European Association for the Study of the Liver–Chronic Liver Failure Consortium and the Chinese Group on the Study of Severe Hepatitis B. Individuals were excluded if they had hepatocellular carcinoma or other malignancies, autoimmune liver diseases (e.g., autoimmune hepatitis or rheumatic disorders), non-HBV-related hepatitis (including alcohol-, drug-, or virus-induced), or were receiving glucocorticoid or immunosuppressive therapy.

Detailed demographic and clinical characteristics of subjects included in the liver RNA-sequencing cohort (HC, n = 14; LC, n = 17; ACLF, n = 18) are provided in **Supplementary Table S1**. Corresponding data for the validation cohort (HC, n = 5; LC, n = 5; ACLF, n = 5) are presented in **Supplementary Table S2**.

### ACLF mouse model

Specific pathogen-free grade C57BL/6J wild-type mice were purchased from Beijing HFK Bio-Technology Co., Ltd., and housed at the Animal Center of the Shanghai Institute of Materia Medica, Chinese Academy of Sciences, under standard Specific pathogen-free conditions. All animal experiments were conducted in accordance with the institutional guidelines for animal care and were approved by the Institutional Animal Care and Use Committee (IACUC) of the Shanghai Institute of Materia Medica (approval numbers 2025-08-LC1-60). Mice were maintained under a 12-hour light/dark cycle at a temperature of 21–25°C and a relative humidity of 55%, with 12–16 air changes per hour. Animals had ad libitum access to commercially available standard chow and water. Eight-week-old male C57BL/6J mice were used to establish the ACLF model following a previously described protocol (40). Briefly, mice received intraperitoneal injections of CCl_4_ at 0.2 mL/kg twice per week for 8 weeks, followed by a single administration of a double dose (0.4 mL/kg), and were then intraperitoneally challenged with *Klebsiella pneumoniae*. For Mitoquinone mesylate (MitoQ) treatment, mice were administered MitoQ at a dose of 5 mg/kg dissolved in phosphate-buffered saline (PBS) via intraperitoneal injection. MitoQ was given three times: 12 h before and 12 h after the double-dose CCl_4_ injection, and 2 h prior to *Klebsiella pneumoniae* challenge.

### Mouse serum assay and liver function analyses

ALT and TBIL levels were measured in animals to evaluate liver injury on a Hitachi 7020 automatic analyzer (Hitachi, Tokyo, Japan) according to the manufacturer’s instructions.

### mRNA sequencing

Liver tissues from human subjects in the HC (n = 14), LC (n = 17), and ACLF (n = 18) groups, as well as mouse liver samples from the HC (n = 4), Fibrosis (n = 4), and ACLF groups at 0 h (n = 19), 48 h (n = 5), 60 h (n = 5), 72 h (n = 5), and 84 h (n = 4), were included in this study. All specimens were collected in advance and stored at −80 °C. For RNA extraction, approximately 50 mg of frozen liver tissue was processed under liquid nitrogen and homogenized using grinding beads. Total RNA was isolated using the FastPure^®^ Cell/Tissue Total RNA Isolation Kit V2 (Vazyme, China) according to the manufacturer’s protocol. RNA purity was determined using a NanoDrop spectrophotometer based on OD260/280 and OD260/230 ratios, while RNA integrity was evaluated using Agilent 2100/4150.

mRNA sequencing libraries were prepared using the TruSeq RNA LT Sample Prep Kit v2 (Illumina, San Diego, CA, USA). mRNA was enriched from total RNA using Oligo(dT) magnetic beads based on polyA selection and then fragmented using divalent cations. First-strand cDNA was synthesized using random hexamer primers and reverse transcriptase, followed by second-strand cDNA synthesis. The double-stranded cDNA underwent end-repair, A-tailing, and adapter ligation. After size selection (targeting 200–300 bp fragments) and PCR amplification, the final libraries were quantified using Qubit 2.0 and validated for insert size. Sequencing was performed on the Illumina platform in paired-end 150 bp mode.

### Differential gene expression analysis

Raw sequencing data in FASTQ format were processed using cutadapt to remove adapter sequences and low-quality reads (length < 50 nt, > 10% unknown bases, or Qphred < 5). For the human cohort, clean reads were aligned to the GRCh38 (GENCODE v47) reference genome using STAR (default parameters). For the mouse cohort, reads were mapped to the mm10 (GRCm38) reference genome using HISAT2.

Gene-level quantification was performed using featureCounts. To ensure inter-sample comparability, raw counts were normalized to FPKM. Specifically, gene lengths were extracted from corresponding GTF annotations (GENCODE v47 for human and GENCODE vM36 for mouse). For genes with multiple isoforms, the symbol with the highest mean count was retained to resolve duplicate entries. Differential expression analysis was then conducted using the edgeR package in R (v.4.3.1). The edgeR package was applied to identify differentially expressed genes (DEGs) among comparison groups. Principal component analysis (PCA) and visualization of DEG distributions were conducted using the ggplot2 package.

### Gene ontology enrichment analysis

Gene Ontology (GO) analysis was performed to predict and visualize changes in biological processes associated with DEGs exhibiting distinct expression patterns. The top 300 enriched biological processes, ranked by p-value, were identified for each comparison group and categorized as major functional contributors to ACLF progression, including inflammation, immune response, metabolism, coagulation, wounding, response to stimulus, and related processes. The number of biological processes corresponding to each functional category was visualized using ggplot2 and GraphPad Prism (v.8.0.2). Associations among the top 30 enriched biological processes in each comparison group were visualized using Cytoscape (v.3.10.1).

### KEGG pathway and gene set enrichment analysis

KEGG pathway analysis was conducted using gene set enrichment analysis (GSEA). Pathway modules associated with ACLF progression, including metabolism, transport and catabolism, the endocrine system, the digestive system, and endocrine and metabolic diseases, were analyzed. Normalized Enrichment Scores (NESs) were calculated to evaluate the degree of pathway enrichment. For both human and mouse datasets, pathways significantly enriched across all three comparison groups were further examined. In addition, pathways consistently enriched in all three human comparison groups were selected for visualization of gene expression patterns within these pathways using ggplot2.

### Temporal expression pattern and clustering analysis

Genes displaying similar expression patterns across disease progression stages from HC to LC (or fibrosis) to ACLF were subjected to GO biological process enrichment analysis. Temporal gene expression dynamics during ACLF induction in mice were further analyzed using the mfuzz clustering algorithm to identify stage-specific biological process enrichment.

### Immune-related gene expression and cell abundance estimation

Changes in the expression levels of cytokines and major histocompatibility complex (MHC) molecules during ACLF progression were visualized using the pheatmap R package. The relative abundance of immune cell populations in each sample was estimated using MCPcounter for human datasets and mMCPcounter for mouse datasets.

### Protein–protein interaction network analysis

Protein–protein interaction networks among differentially expressed genes were constructed using the STRING database to explore potential functional associations and regulatory interactions.

### Visualization of key differential genes

GraphPad Prism (v.8.0.2) was used to visualize the expression levels of key differential genes across different comparison groups.

### RT-qPCR for gene expression analysis

Total RNA was extracted from liver tissues using a Rapid RNA Extraction Kit (Vazyme, #RC112-01) according to the manufacturer’s instructions. For reverse transcription, 1 μg of total RNA was mixed with 4 μL of 5×HiScript II qRT SuperMix and RNase-free water was added to a final volume of 20 μL. The RNA input ranged from 1 pg to 1 μg. The resulting cDNA was used as a template for quantitative PCR (qPCR). Each qPCR reaction contained 5 μL of 2×ChamQ SYBR qPCR Master Mix, 0.2 μL of forward and reverse primers (10 μM), and 1 μL of cDNA template, with double-distilled water (ddH_2_O) added to a final volume of 10 μL. Samples were loaded onto a real-time PCR system, and amplification data were collected to determine the relative expression levels of target genes. Gene expression was calculated based on the amplification curves and normalized to internal control genes.

### Protein concentration determination by BCA assay

Protein concentrations were determined using the Pierce™ bicinchoninic acid (BCA) Protein Assay Kit (Thermo Scientific) according to the manufacturer’s instructions. Briefly, BCA working reagent was freshly prepared by mixing Reagent A and Reagent B at a 50:1 ratio. Samples and standards were added to a 96-well plate (25 µL per well), followed by the addition of 200 µL of working reagent. After thorough mixing, the plate was incubated at 37°C for 30 min. Absorbance was measured at 562 nm using a multifunctional microplate reader. Protein concentrations were calculated based on the BSA standard curve.

### Western blot analysis

Cell and tissue lysates were subjected to electrophoresis through SDS-PAGE and transferred to Nitrocellulose (NC) or polyvinylidene difluoride (PVDF) membranes, and then blotted with antibodies. Target protein bands were visualized using the enhanced chemiluminescence method in a ChemiScope3400 imaging system using ECL substrate (Clinx). Primary antibodies used are listed in antibodies section of **Supplementary Materials**. Primary antibodies used included: COX IV (Cell Signaling Technology, #4850), CPT1A (Abcam, #ab128568), GLUD1 (Cell Signaling Technology, #12793), PDHA1 (Cell Signaling Technology, #3205), TREM2 (Cell Signaling Technology, #55739), and CCR2 (Affinity Biosciences, #DF2711).

### ATP measurement

Hepatic adenosine triphosphate (ATP) levels were measured using an Enhanced ATP Assay Kit (Beyotime, S0027) according to the manufacturer’s protocol. Briefly, approximately 20 mg of human or mouse liver tissue was homogenized in ATP lysis buffer on ice and centrifuged at 12,000 × g for 5 min at 4°C. The supernatant was collected for ATP determination. ATP working solution was added to a white 96-well plate, followed by the addition of samples or ATP standards. Luminescence was detected using a multifunctional microplate reader. ATP concentrations were calculated based on the standard curve and normalized to protein concentrations determined by the BCA assay.

### Statistical analysis

Statistical analyses were performed using GraphPad Prism (version 8.0.1) and R (version 4.3.1). A log-rank test was used to compare differences in mortality rates between groups. Normality was assessed by Shapiro-Wilk test. For two-group comparisons, Student’s t test or Welch’s t test was used for normally distributed data with equal or unequal variances, respectively; the Mann-Whitney U test was used for non-normal data. For three or more groups, one-way ANOVA with Tukey’s *post hoc* test, Welch’s ANOVA, or Kruskal-Wallis test was applied based on data distribution and variance homogeneity. Bar plots are shown as mean ± SD. Correlations were assessed using Pearson’s r or Spearman’s ρ. Statistical significance was denoted by asterisks in the figures: *P < 0.05; **P < 0.01, ***P < 0.001, ****P < 0.0001.

## Result

### Transcriptomic profiles of liver tissues from ACLF patients and mice

**Figure 1:**
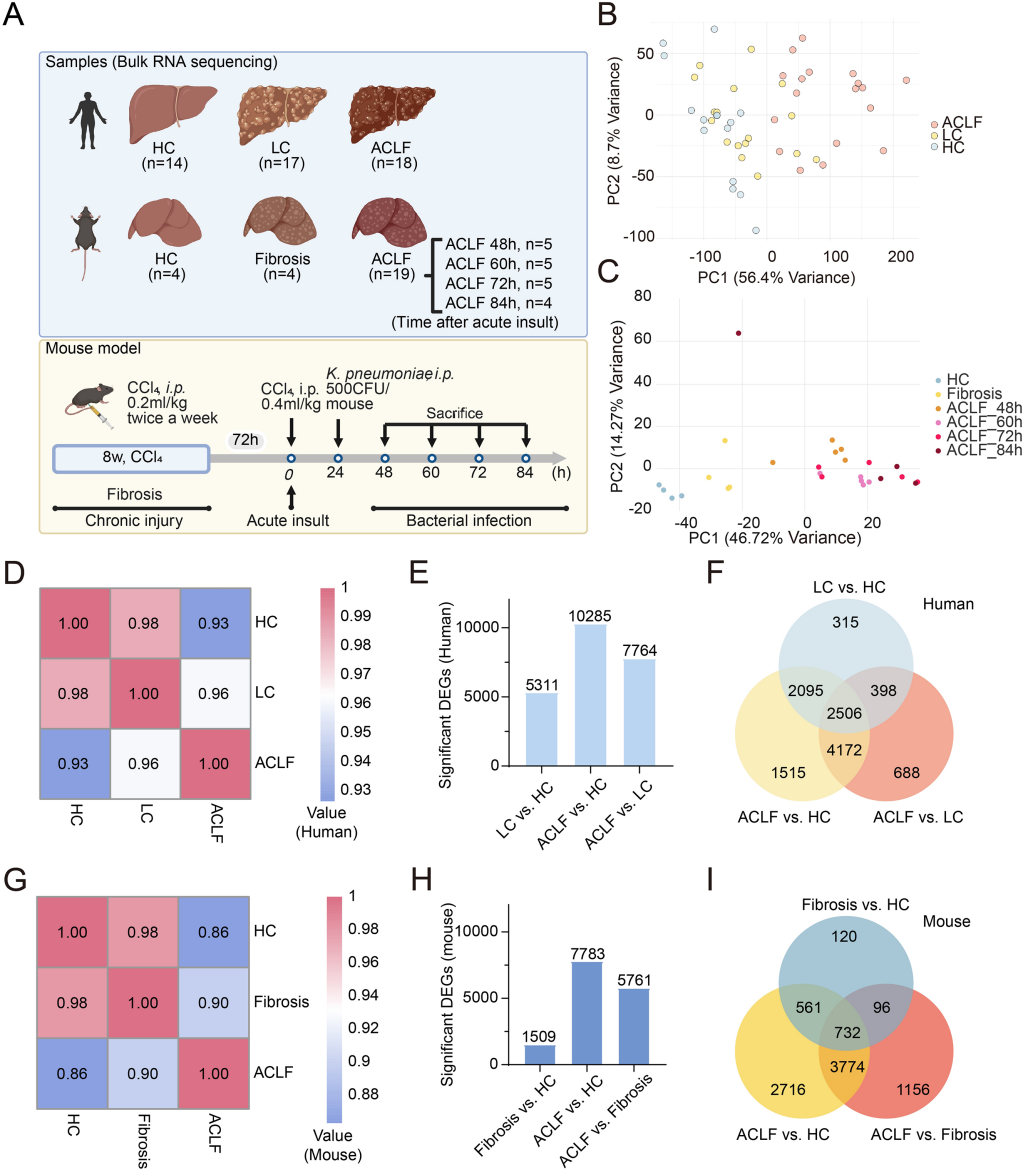
Overview of human and mouse liver transcriptomic profiling across ACLF progression. **(A)** Schematic of bulk RNA-seq sample grouping and mouse ACLF modeling strategy. Human liver samples included HC, LC and ACLF patients. Mouse samples included HC, fibrosis, and ACLF groups at multiple timepoints post-injury. **(B, C)** Principal component analysis plots of liver transcriptomes in humans **(B)** and mice **(C)**, showing distinct transcriptomic shifts across disease stages. **(D)** Correlation heatmap showing pairwise transcriptomic similarity between human groups (HC, LC, ACLF). **(E)** Bar plot showing the number of DEGs identified between human groups. **(F)** Venn diagram illustrating overlap of DEGs among the three group comparisons in human liver samples. **(G)** Correlation heatmap showing transcriptomic similarity across mouse groups (HC, Fibrosis, ACLF). **(H)** Bar plot showing the number of DEGs between mouse groups. **(I)** Venn diagram displaying DEG overlap across Fibrosis vs. HC, ACLF vs. HC, and ACLF vs. Fibrosis comparisons in mouse livers.

To address the critical knowledge gap in molecular mechanisms driving acute-on-chronic liver failure (ACLF), we performed comprehensive transcriptomic profiling of liver tissues from clinical cohorts and experimental mouse models. The experimental design and cohort composition for transcriptomic sequencing were summarized in **Figure 1A**. Human cohorts included healthy controls (HC), patients with liver cirrhosis (LC), and those with HBV-associated ACLF, while mouse cohorts comprised healthy, fibrotic, and ACLF-modeled mice. Clinical characterization of the patient cohort (**Supplementary Table S1**) demonstrated that ACLF patients exhibited significantly elevated HBV DNA, along with markedly increased levels of alanine aminotransferase, aspartate aminotransferase, total bilirubin, and international normalized ratio, compared to LC patients (**Supplementary Table S1**).

Principal component analysis of hepatic transcriptomes revealed distinct disease-stage clustering in both human and mouse models. Human samples segregated into three discrete groups (HC, LC, ACLF) along a progressive disease trajectory (HC→LC→ACLF; **Figure 1B**). A similar pattern was observed in mouse liver transcriptomes: samples within the ACLF group clustered tightly and were clearly distinct from those of the fibrosis and HC groups, which were aligned along a comparable axis (**Figure 1C**). Notably, one outlier sample from the 84-hour ACLF murine cohort was excluded from subsequent analyses due to pronounced deviation from intra-group clustering (**Figure 1C**).

Cross-group correlation analyses demonstrated strong concordance among biological replicates within each condition, while lower correlations were observed between experimental groups, suggesting robust transcriptomic divergence across disease stages both in patients and mice (**Figures 1D, G**, **Supplementary Figures S1A, B, S2A**). Multi-group DEGs analysis identified stage-specific gene signatures, with intersection analysis revealing conserved dysregulated pathways between human ACLF and mouse models (**Figures 1E, F, H, I**, **Supplementary Figure S2B**). These cross-species transcriptional regulators provide critical insights into ACLF pathogenesis and establish a foundation for dissecting the molecular mechanisms underlies disease progression.

### Prominent dysregulation of inflammatory signaling, immune response, and metabolism in the livers of HBV-ACLF patients and mouse models

**Figure 2:**
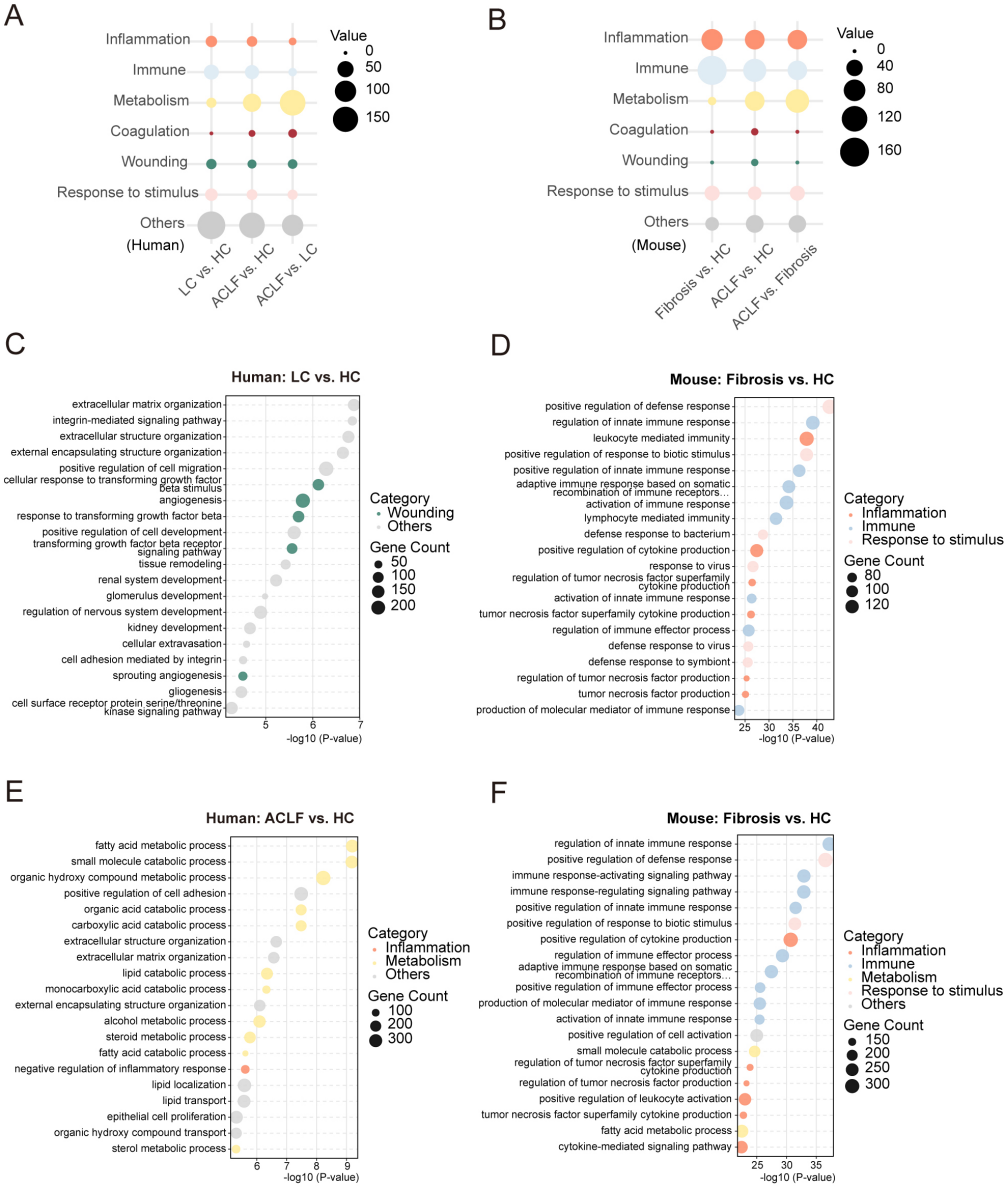
Functional classification and GO enrichment analysis of DEGs in human and mouse livers during ACLF progression. **(A)** Bubble plot summarizing the distribution of the top 300 GO biological process terms by functional category in human liver comparisons (LC vs. HC, ACLF vs. HC, ACLF vs. LC). **(B)** Bubble plot summarizing the distribution of the top 300 GO biological process terms in mouse liver comparisons (Fibrosis vs. HC, ACLF vs. HC, ACLF vs. Fibrosis). **(C)** Enrichment GO pathway of representative top 20 biological process terms in the LC vs. HC comparison of human liver samples. **(D)** Enrichment GO pathway of representative top 20 biological process terms in the Fibrosis vs. HC comparison of mouse liver samples. **(E)** Enrichment GO pathway of representative top 20 biological process terms in the ACLF vs. HC comparison of human liver samples. **(F)** Enrichment GO pathway of representative top 20 biological process terms in the ACLF vs. HC comparison of mouse liver samples.

To delineate the dynamic molecular shifts underlying ACLF progression, we performed functional synergy analysis of resulting DEGs (LC/liver fibrosis vs. HC, ACLF vs. HC, ACLF vs. LC/liver fibrosis) revealed stage-specific biological perturbations in both human and mouse cohorts. Top 300 enriched biological processes were further categorized into seven pathophysiological themes enriched across ACLF progression: inflammation, immunity, metabolism, coagulation, tissue injury, stimulus response (including microbial and non-microbial stimuli), and other unclassified processes (**Figures 2A, B**). Strikingly, although the number of immune and inflammatory pathways declined, they consistently converged on specific pathways throughout ACLF progression in both species. In contrast, metabolic processes were persistently altered across all stages and demonstrated the most extensive dysregulation in ACLF, highlighting their dominant role in liver functional deterioration (**Figures 2A, B**). In addition, coagulation-related signatures were markedly amplified in human ACLF compared to LC. However, no clear distinction in wound-associated pathways was observed. This pattern diverged in mice due to reduced representation of coagulation terms among top-ranked pathways (**Figures 2A, B**). Response-to-stimulus processes showed mild downregulation in human ACLF but remained stable in mice, suggesting species-specific modulation of stress adaptation mechanisms (**Figures 2A, B**). Collectively, these findings highlight inflammatory decline, immune suppression, and metabolic collapse as central drivers of ACLF pathogenesis.

GO similarity analysis of the top 20 enriched biological processes in each pairwise comparison revealed distinct stage-dependent pathway activation (**Figures 2C–F**, **Supplementary Figures S3A, B**). In LC patients, transcriptional profiles were dominated by tissue remodeling and fibrogenesis (e.g., extracellular matrix organization, integrin-mediated signaling, tissue remodeling), combined with tissue injury (e.g., angiogenesis, response to transforming growth factor beta) (**Figure 2C**). While fibrosis-stage mice exhibited robust inflammation (e.g., leukocyte mediated immunity, positive regulation of cytokine production), immunity (e.g., activation of immune response, activation of innate immune response), and responses to external stimuli pathway activation (e.g., positive regulation of response to biotic stimulus), reflecting an earlier inflammatory phase compared to LC patients (**Figure 2D**). Furthermore, human ACLF livers were characterized by metabolic decompensation (fatty acid, steroid, and alcohol metabolism) alongside inflammatory resolution (negative regulation of inflammatory response) (**Figure 2E**). Conversely, ACLF mice maintained heightened immune-inflammatory activity (positive regulation of cytokine production, positive regulation of leukocyte) superimposed on metabolic perturbations (fatty acid catabolism; **Figure 2F**). This divergence suggests unresolved inflammation in mouse ACLF may model early decompensation phases, whereas human ACLF represents a late stage dominated by metabolic failure.

Intersection analysis of ACLF-associated DEGs across species revealed conserved disruption of core metabolic programs (fatty acid metabolism, small molecule catabolism, amino acid metabolism; **Supplementary Figures S3A, B**). These conserved signatures highlight metabolic disorder as a non-redundant hallmark of ACLF, independent of species-specific inflammatory contexts. Overall, our multi-layered transcriptomic dissection identifies tripartite dysregulation of inflammation, immune response, and metabolism as the pathogenic cornerstone of ACLF. The progressive transition from fibro-inflammatory activation to metabolic collapse in humans, contrasted with sustained inflammation in mouse ACLF, underscores critical phase-specific therapeutic windows. Conserved metabolic disruptions across species highlight promising targets for functional restoration in ACLF management.

### Stage-specific transcriptomic clustering reveals conserved immune-metabolic reprogramming across species in ACLF

**Figure 3:**
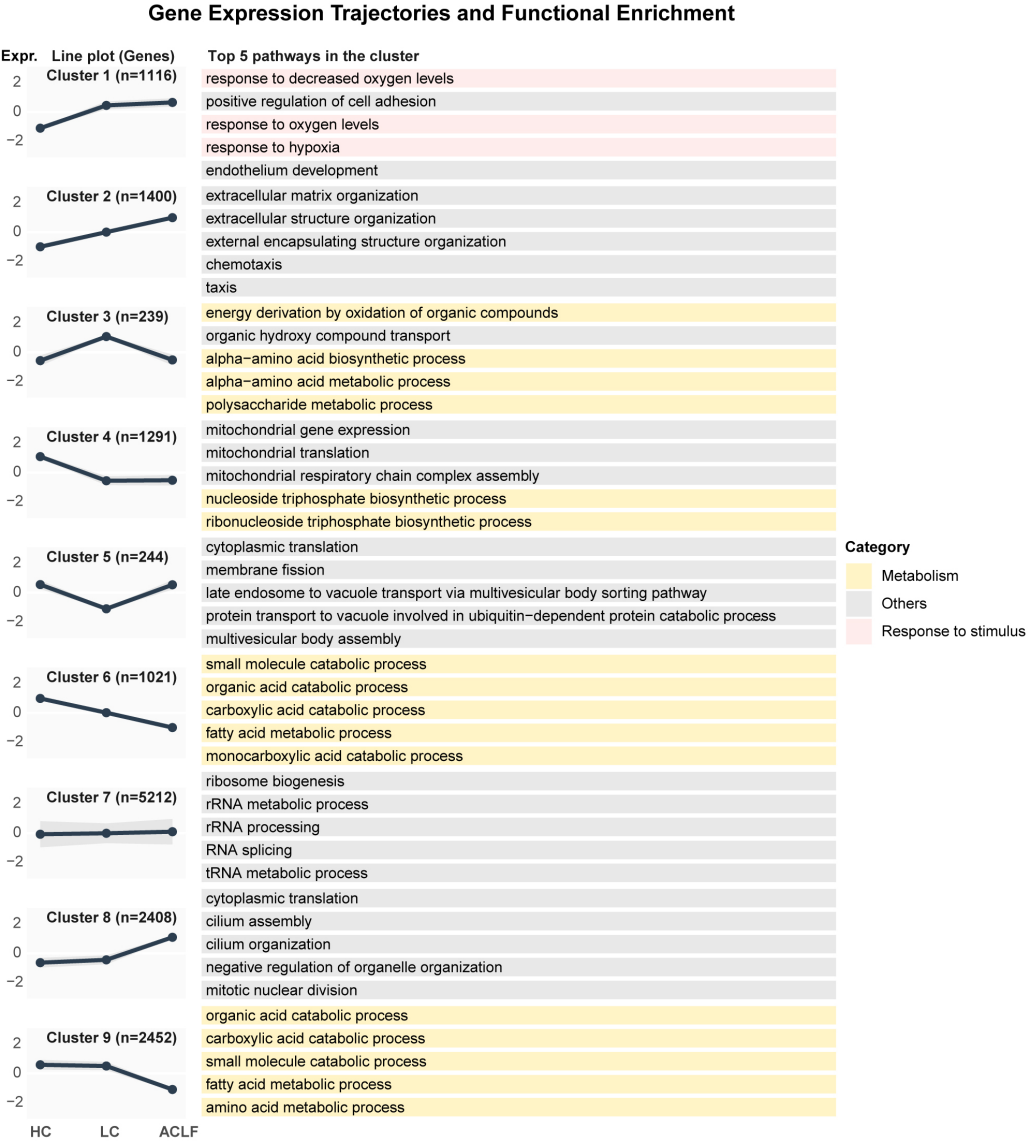
Z-score normalized gene expression profiles (9 clusters) and functional enrichment analysis in human liver samples. Left panel: The solid black line indicates the mean expression level of all genes in the cluster across disease stages (HC, LC, ACLF), while the shaded ribbon represents the standard deviation. Right panel: Representative top enriched GO biological processes for each cluster. The colored bars indicate the functional category of the GO terms, matching the color scheme in **Figure 2**.

To further resolve dynamic transcriptional reprogramming during ACLF progression, we performed comparative gene clustering using liver transcriptomes from human cohorts (HC, LC, ACLF) and mouse models (HC, fibrosis, ACLF). This analysis revealed nine conserved gene clusters (Clusters 1–9) with stage-dependent expression trajectories (**Figure 3**, **Supplementary Figure S4**). These clusters revealed distinct stage-associated transcriptional trajectories that were largely conserved across species.

In humans, immune-fibrotic activation dominated ACLF-associated clusters: Cluster 1 (hypoxia response, leukocyte adhesion) and Cluster 2 were sharply upregulated in ACLF, reflecting enhanced immune activity and fibrotic remodeling (**Figure 3**). Conversely, metabolic clusters (Clusters 3, 4, 6, 9) exhibited progressive dysregulation (**Figure 3**). Notably, Cluster 4, enriched for mitochondrial energy metabolism (respiratory chain complex assembly, ATP biosynthesis), collapsed early in LC and remained suppressed through ACLF, while Clusters 3, 6, and 9 declined progressively from LC to ACLF (**Figure 3**). These clusters were enriched for amino acid metabolism (urea cycle, TCA cycle), lipid catabolism (fatty acid β-oxidation, purine degradation), and sterol detoxification (cholesterol, steroid, alcohol metabolism), marking systemic metabolic failure (**Figure 3**). Additional transcriptional modules showed stage-specific modules: Cluster 7, linked to ribosomal biogenesis and translational control, peaked in LC and declined in ACLF, whereas Cluster 8, involved in mitotic processes and cilium assembly, was uniquely activated in ACLF, potentially reflecting aberrant regenerative attempts under injury (**Figure 3**). Together, these findings delineate a coordinated trajectory of immune activation, metabolic collapse, and dysfunctional regeneration during ACLF progression.

Cross-species alignment of clusters revealed striking functional conservation (**Supplementary Figure S4**). Immune-related clusters (human Clusters 1–2; mouse Clusters 1–2) showed parallel upregulation in ACLF, underscoring conserved immune activation across species (**Supplementary Figure S4**). Metabolic clusters (human Cluster 3; mouse Clusters 3 and 6) displayed transient upregulation at intermediate stages (LC/fibrosis) followed by ACLF suppression, indicating transient metabolic activation followed by exhaustion (**Supplementary Figure S4**). Protein folding and proteostasis clusters (Cluster 5 in both species) were downregulated in ACLF, suggesting impaired stress responses. Translational regulation (Cluster 7) and energy metabolism (human Clusters 6 and 9; mouse Clusters 4 and 9) similarly showed declining trends in ACLF (**Supplementary Figure S4**). Interestingly, Cluster 8-related to cell cycle control-was consistently induced in ACLF in both species (**Supplementary Figure S4**). These overlaps validate murine ACLF as a translational model capturing core human pathophysiology.

To further dissect the temporal regulatory dynamics, we conducted longitudinal RNA-seq in murine ACLF (48 h, 60 h, 72 h, 84 h post-induction) and seven clusters were defined (**Supplementary Figure S5**). Early-phase immune activation dominated Clusters 1 and 4 enriched in T cell regulation, leukocyte adhesion, antigen presentation, and cytokine signaling (**Supplementary Figure S5**), while Cluster 3 adaptive immunity (B/T cell proliferation) and Cluster 5 innate immunity (phagocytosis and pattern recognition) were transiently upregulated, peaking at 60 h (**Supplementary Figure S5**). Cluster 7 (interferon-related pathways), peaking between 60–72 h, marked a mid-stage antiviral response, followed by paradoxical late-phase suppression of antiviral defense (Clusters 4, 5 and 7 after 72 h) despite persistent inflammation (**Supplementary Figure S5**). Conversely, metabolic Cluster 6 (fatty acid and organic acid metabolism) showed continuous decline, mirroring human ACLF progression (**Supplementary Figure S5**). This temporal cascade-early immune priming (48 h), mid-phase inflammatory amplification (60–72 h), and late immune-metabolic collapse (84 h)-recapitulates human ACLF progression, revealing a conserved “inflammatory exhaustion” phenotype.

Taken together, our multi-dimensional analysis uncovers a unified ACLF progression framework: early phase (LC/fibrosis) with immune priming and transient metabolic activation, mid phase (LC→ACLF transition) with inflammatory amplification and metabolic decompensation, and late-phase collapse of metabolic and immune homeostasis. These results provide mechanistic insight into ACLF pathogenesis and offer a basis for defining stage-specific therapeutic strategies.

### Cross-species metabolic pathway enrichment reveals immune-associated activation coupled with global suppression of mitochondrial-centered metabolism during ACLF progression

**Figure 4:**
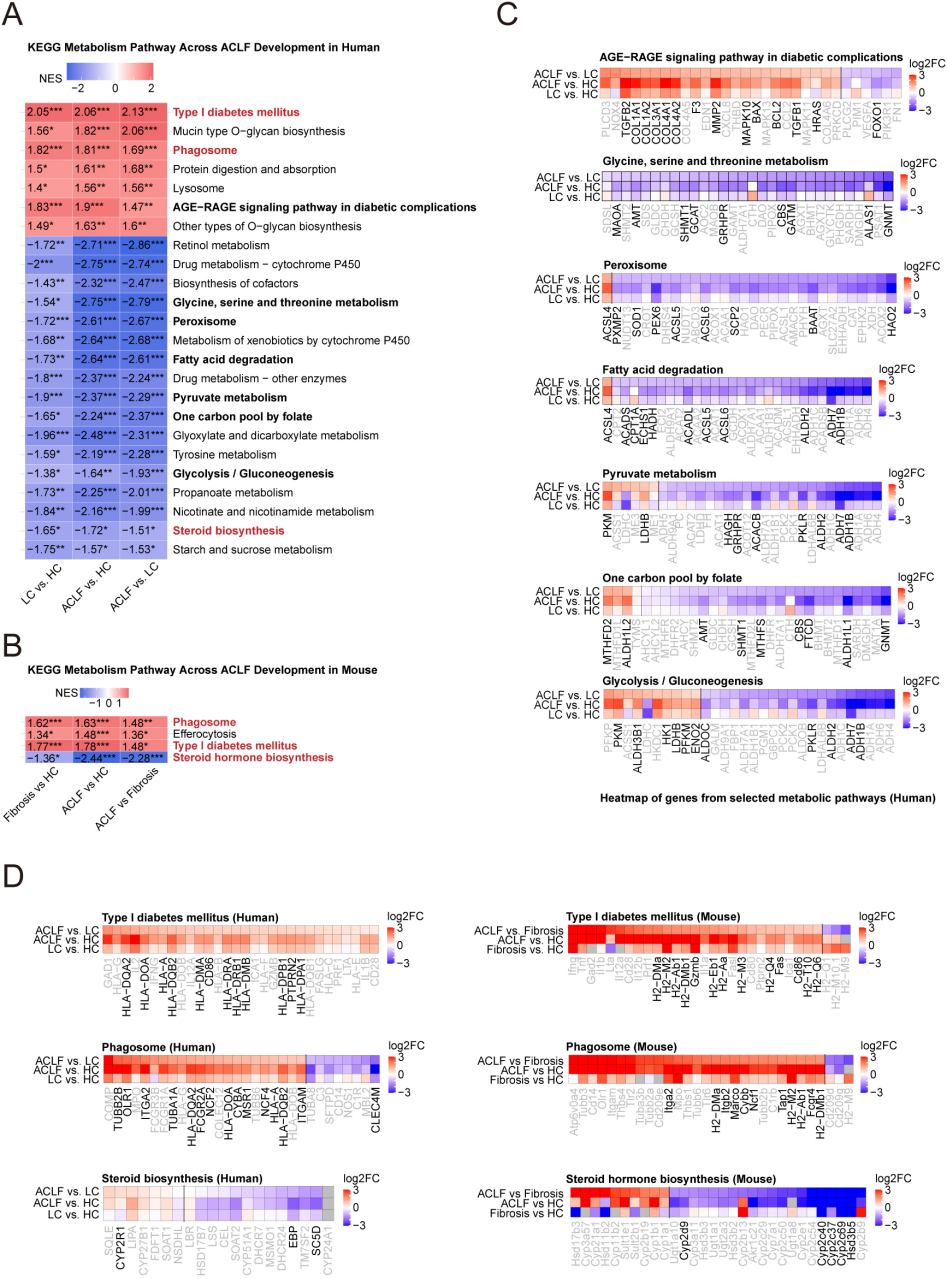
KEGG pathway analysis of metabolism-related pathways in human and mouse liver transcriptomes. **(A)** Heatmap showing normalized enrichment scores of significantly enriched KEGG metabolism-related pathways across three human liver comparisons (LC vs. HC, ACLF vs. HC, ACLF vs. LC). Pathways labeled in red are shared with mouse data in **(B)**; those in black are further explored in **(C)**. **(B)** Heatmap showing Normalized Enrichment Score values for significantly enriched KEGG metabolism-related pathways across three mouse liver comparisons (Fibrosis vs. HC, ACLF vs. HC, ACLF vs. Fibrosis). Red-labeled pathways are conserved across species. **(C)** Heatmaps showing top 30 DEGs in selected KEGG pathways from **(A)**. Black gene names represent DEGs significantly altered in all three human comparisons; gray gene names denote partial or non-significant changes. **(D)** Comparative heatmaps of top 30 DEGs in metabolism-related pathways commonly enriched in both human and mouse datasets from **(A, B)**. Black gene names indicate consistent significance across all three comparisons.

To elucidate hepatic metabolic reprogramming during ACLF progression, we performed systematic KEGG pathway analysis of liver transcriptomes and identified 26 human and 9 murine metabolic pathways consistently altered across ACLF progression (**Figures 4A, B**). Cross-species overlap highlighted three pathways-Type I diabetes mellitus, Phagosome, and Steroid biosynthesis-as central to ACLF pathogenesis, implicating immune-metabolic crosstalk and steroidogenic failure in disease evolution (**Figures 4A, B**).

Gene-centric analysis of key metabolic pathways uncovered systemic dysfunction across critical hepatic processes in ACLF (**Figure 4C**). In the glycine, serine and threonine metabolism pathway, downregulation of GNMT, MAT1A, and CBS-central regulators of S-adenosylmethionine synthesis-indicated disruption of methyl donor metabolism, impairing epigenetic regulation and detoxification. In fatty acid degradation, concurrent suppression of ADH4, CPT1A, and ACOX1 revealed defective β-oxidation, compounded by diminished LDHD and ALDH3B1 expression in pyruvate metabolism pathway, reflecting collapse of carbohydrate catabolism (**Figure 4C**). Meanwhile, these perturbations collectively drove hepatic functional failure. The downregulation of pyruvate metabolism and gluconeogenesis reflects compromised glucose production. Disruption of the one carbon pool by folate may affect methylation and translational regulation (**Figure 4C**). Suppression of retinol metabolism indicates reduced vitamin A storage and antioxidant function, while diminished lipid oxidation and fatty acid degradation imply impaired energy production, which are the hallmarks of terminal hepatic failure in ACLF patients (**Figure 4C**).

Gene-level visualization revealed parallel immune-metabolic dysfunction in both species (**Figure 4D**). While Type I diabetes mellitus and Phagosome pathways exhibited conserved upregulation of immune hubs (HLA-DRA, TUBB in humans; H2-Aa, CYBA in mice), steroidogenic pathways were uniformly suppressed, marked by downregulation of CYP21A2 (human steroid biosynthesis) and Cyp2a4 (murine steroid metabolism) (**Figure 4D**). The coexistence of immune hyperactivation and steroidogenic failure suggests a disconnect between the endocrine and immune systems, which worsens metabolic collapse.

Comprehensive analysis of metabolic pathways altered between disease stages identified 68 dysregulated pathways in humans and 52 in mice, with significant cross-species overlap in lipid metabolism (e.g., fatty acid degradation, steroid biosynthesis), amino acid metabolism (e.g., glycine, serine and threonine metabolism; arginine and proline metabolism), and nucleotide/cofactor metabolism (e.g., one carbon pool by folate, nicotinate and nicotinamide metabolism) (**Supplementary Figures S6A, B**). Notably, most of these pathways exhibited progressive downregulation from fibrosis to ACLF, confirming pan-metabolic suppression as a hallmark of advanced disease. While murine ACLF faithfully recapitulated human terminal metabolic failure, evidenced by concordant suppression of fatty acid degradation, pyruvate metabolism, and steroid biosynthesis, species-specific divergence emerged in early stages (**Supplementary Figures S6A, B**). Murine liver fibrosis showed attenuated metabolic disruption compared to human cirrhosis, potentially reflecting model limitations in replicating early adaptations. Notably, murine-specific activation of AGE-RAGE signaling, absent in human cohorts, likely stems from differential etiologies (diet-induced vs. viral injury) (**Supplementary Figures S6A, B**). Comprehensive comparison revealed that despite species differences, both human and murine ACLF at the terminal stage exhibit a consistent pattern of metabolic downregulation, characterized by coordinated suppression of multiple mitochondria-centered energy metabolism pathways, including pyruvate metabolism, the tricarboxylic acid cycle, fatty acid oxidation, and oxidative phosphorylation.

**Figure 5:**
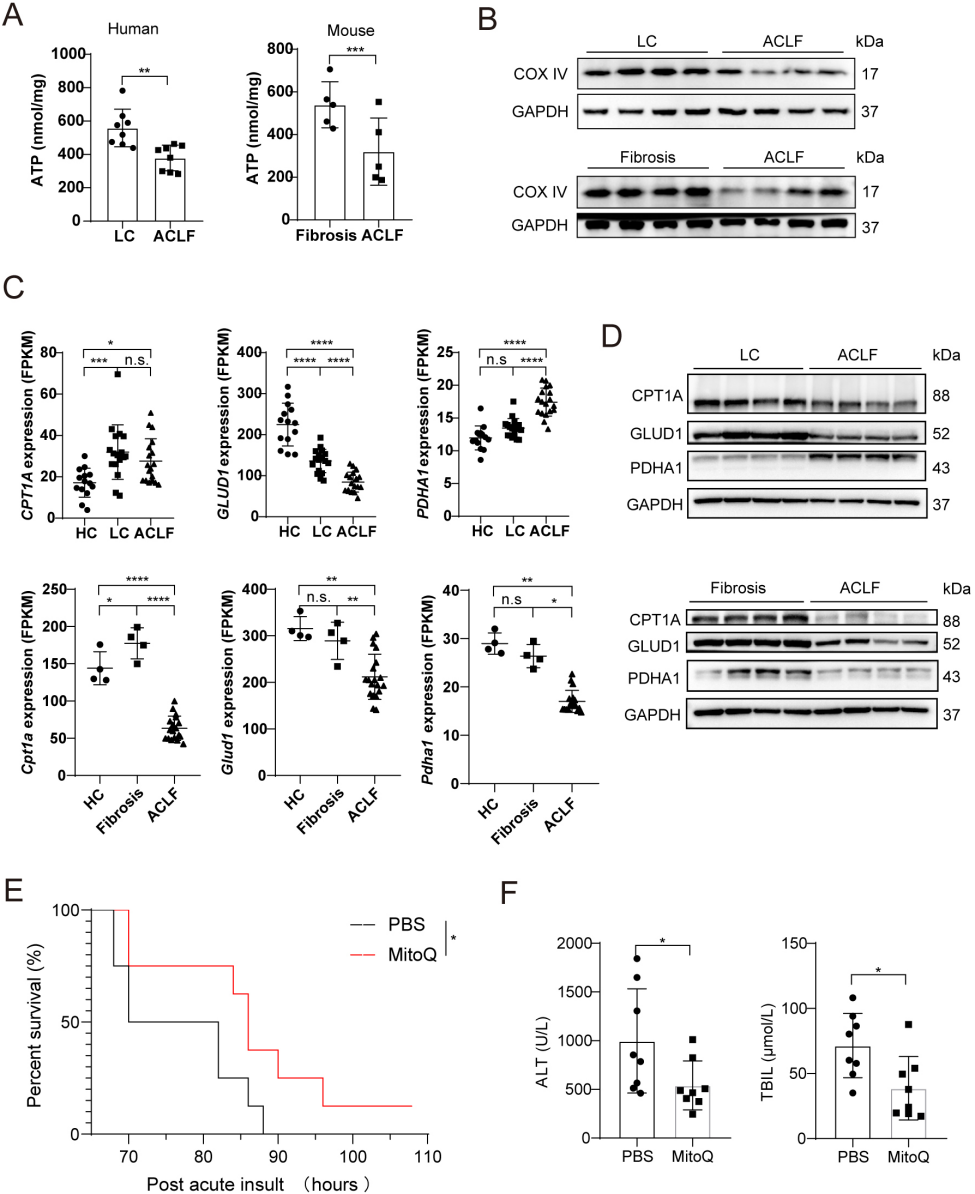
Metabolic Dysfunction-Associated Impairment of Mitochondrial Anaplerotic Capacity in ACLF. **(A)** Hepatic ATP levels in LC/fibrosis and ACLF samples. **(B)** Western blot analysis reveals protein levels of COX IV in liver tissues from human LC and ACLF patients and from mouse fibrosis and ACLF models. **(C)** RNA sequencing analysis of key metabolic genes (*CPT1A/Cpt1a*, *GLUD1/Glud1*, *PDHA1/Pdha1*) in human (HC, LC, ACLF) and mouse (HC, fibrosis, ACLF) liver samples. **(D)** Western blot of CPT1A, GLUD1, and PDHA1 protein expression in human and mouse liver tissues. **(E)** Kaplan-Meier survival curves of ACLF mice treated with PBS or the mitochondrial antioxidant MitoQ. **(F)** Serum ALT and TBIL levels in ACLF mice following treatment with PBS or MitoQ. Bars represent mean ± SD; ****P < 0.0001, ***P < 0.001, **P < 0.01, *P < 0. 05, n.s., no significance; ALT, alanine aminotransferase; TBIL, total bilirubin.

To further validate the suppression of mitochondrial energy metabolism observed in transcriptomic analyses, we measured ATP content and COX IV protein expression in liver tissues from both ACLF patients and murine models (**Figures 5A, B**). Results demonstrated a significant reduction in ACLF hepatic ATP levels (**Figure 5A**) and decreased COX IV protein abundance (**Figure 5B**) compared to LC or liver fibrosis, indicating impaired mitochondrial function during ACLF progression. Consistent with transcriptomic data, key mitochondrial metabolic regulators CPT1A, GLUD1 and PDHA1 showed decreased protein expression in murine ACLF samples (**Figures 5C, D**). However, in human ACLF, PDHA1 protein was paradoxically upregulated despite the downregulation of CPT1A and GLUD1 (**Figures 5C, D**). This divergent regulation of PDHA1 suggests a compensatory but insufficient attempt to restore mitochondrial pyruvate metabolism in advanced disease stages. Building on these findings, treatment with the mitochondrial targeted antioxidant MitoQ significantly improved survival in ACLF mice (**Figure 5E**), accompanied by reductions in serum alanine aminotransferase (ALT) and total bilirubin (TBIL) levels (**Figure 5F**), supporting its potential to ameliorate mitochondrial dysfunction and liver injury in ACLF.

### Immune landscape characterization reveals monocyte-macrophage expansion and proinflammatory signaling in ACLF

**Figure 6:**
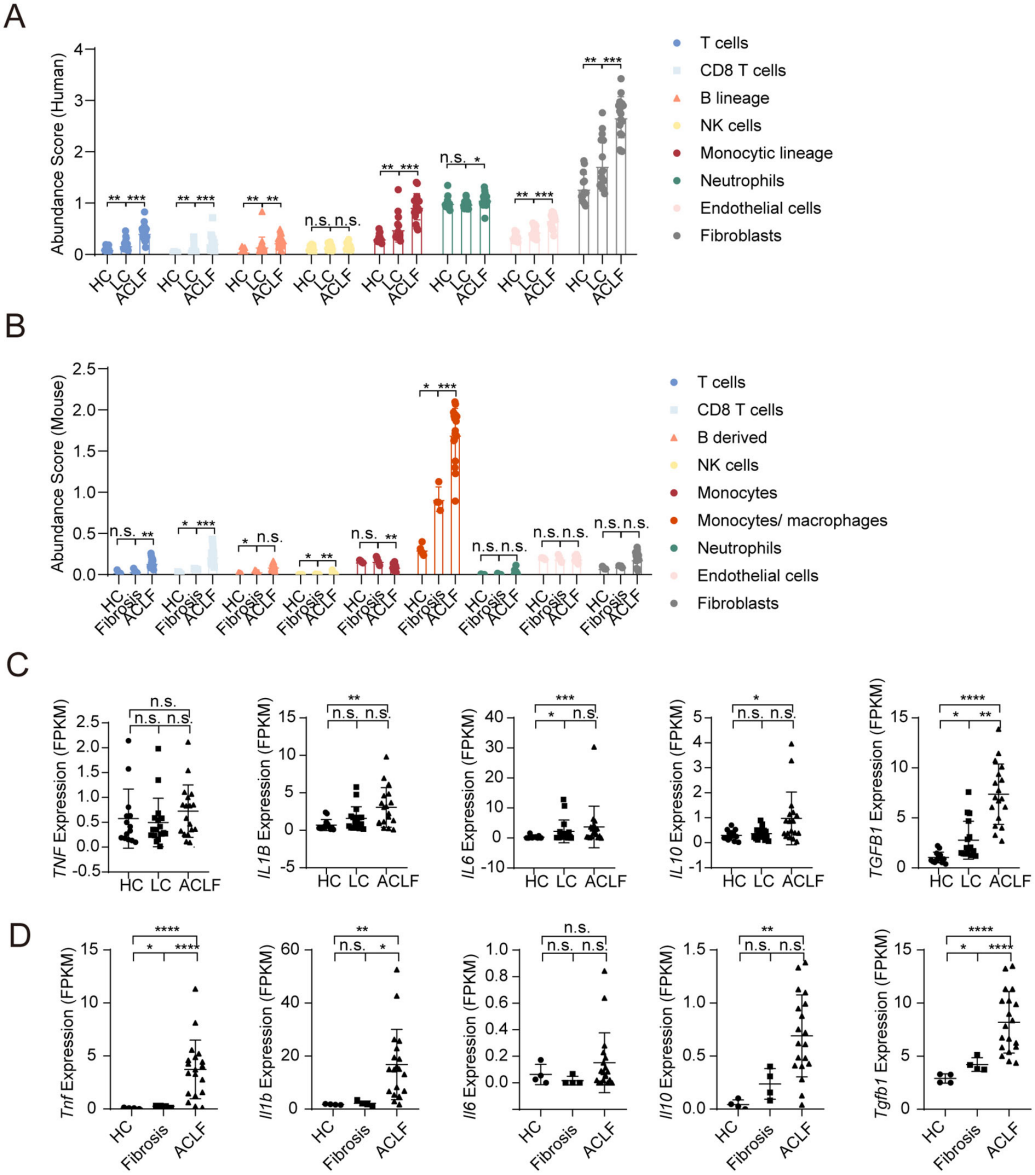
Immune landscape and cytokine expression profiles in human and mouse livers during ACLF progression. **(A)** MCP-counter-based estimation of immune cell infiltration in human liver samples (HC, LC, ACLF). Relative abundance scores are shown for T cells, CD8 T cells, B lineage cells, NK cells, monocyte lineage, neutrophils, endothelial cells, and fibroblasts. **(B)** MCP-counter-based estimation of immune cell infiltration in mouse liver samples (HC, Fibrosis, ACLF). **(C)** Expression of pro-inflammatory (*TNF*, *IL1B*, *IL6*) and anti-inflammatory (*IL10*, *TGFB1*) cytokines in human liver transcriptomes (FPKM values). **(D)** Expression of the same cytokines in mouse liver transcriptomes. Bars represent mean ± SD; ****P < 0.0001, ***P < 0.001, **P < 0.01, *P < 0. 05, n.s., no significance.

Following the comprehensive analysis of metabolic dysregulation, we further examined the immune landscape throughout ACLF progression. MCP-counter-based immune deconvolution revealed pronounced monocyte/macrophage expansion as a hallmark of ACLF across species (**Figures 6A, B**). Human ACLF livers exhibited significantly elevated monocyte/macrophage scores compared to both HC and LC groups (**Figure 6A**). Murine models mirrored this trend, demonstrating exponential monocyte/macrophage accumulation from the fibrosis to ACLF stage (**Figure 6B**). While T/NK cell populations showed species-specific dynamics, modest increases in humans versus marked upregulation in mice, neutrophil infiltration remained stable in both species, suggesting distinct regulatory mechanisms for myeloid versus granulocyte recruitment (**Figures 6A, B**).

To further characterize the immune activation status, we examined the expression of representative pro-inflammatory cytokines (*TNFα*, *IL-1β*, *IL-6*) and anti-inflammatory mediators (*IL-10*, *TGFβ1*) (**Figures 6C, D**). Stage-resolved cytokine profiling uncovered concurrent pro- and anti-inflammatory activation. Human ACLF livers displayed significant upregulation of *IL1B*, *IL6*, *IL10*, *TGFB1*, and variable expression of *TNF* (**Figure 6C**). Mouse ACLF recapitulated this pattern, with parallel elevations in *Tnf*, *Il1b*, *Il6*, *Il10*, and *Tgfb1* (**Figure 6D**). Notably, although TGFβ1 has profibrotic functions, it is generally regarded as immunosuppressive in hepatic inflammation. The co-elevation of pro- and anti-inflammatory cytokines may reflect an immune dysfunction statement under an activated immune background.

Heatmap analysis of cytokine and MHC molecule expression revealed conserved proinflammatory networks in ACLF (**Supplementary Figures S7A, B, S8A, B**). Upregulation of IL1B, IL6, IFNG, and CXCL10 in both species (Human: **Supplementary Figure S7A**; Mouse: **Supplementary Figure S8A**) coincided with enhanced antigen-presentation machinery-notably *HLA-DPA1/DPB1/DRB1* in humans and *H2-Aa/Ab1/Eb1* in mice (Human: **Supplementary Figure S7B**; Mouse: **Supplementary Figure S8B**). This coordinated activation of cytokine signaling and MHC class II expression implies sustained adaptive immune activation through enhanced T cell priming.

Together, ACLF progression is characterized by tripartite immune dysfunction: myeloid-dominated infiltration, cytokine storm with compromised regulatory control, and amplified antigen presentation. The cross-species conservation of these features validates murine ACLF as a clinically relevant model for studying immune-driven liver failure.

### Signature genes identify conserved monocyte-associated markers correlated with disease severity in ACLF

**Figure 7:**
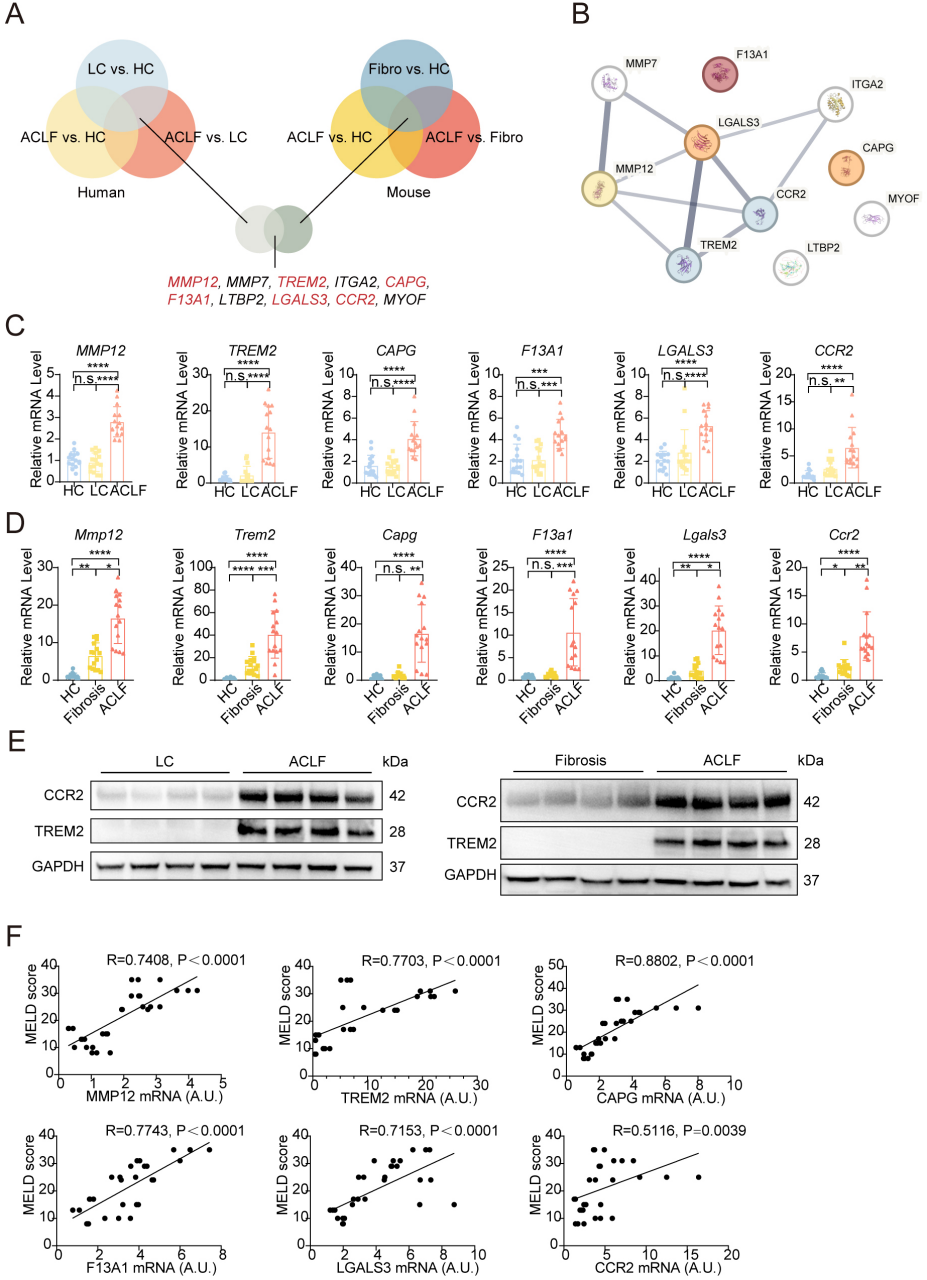
Cross-species identification and validation of monocyte/macrophage-associated genes in ACLF. **(A)** Venn diagram showing the overlap of DEGs across three comparisons in human (LC vs. HC, ACLF vs. HC, ACLF vs. LC) and mouse (Fibrosis vs. HC, ACLF vs. HC, ACLF vs. Fibrosis) liver samples. The top 10 intersecting DEGs are shown, with red-labeled genes associated with monocyte/macrophage functions. **(B)** Protein-protein interaction (PPI) network of the top 10 conserved DEGs as predicted by STRING analysis. **(C)** Validation of the mRNA expression levels of six monocyte/macrophage-associated genes (*MMP12*, *TREM2*, *CAPG*, *F13A1*, *LGALS3*, and *CCR2*) in human liver samples using RT-qPCR. **(D)** Validation of the same six monocyte/macrophage-associated genes in mouse liver samples using RT-qPCR. **(E)** Western blot of TREM2 and CCR2 protein expression in human and mouse liver tissues. **(F)** Correlation analysis between mRNA levels of the six genes and MELD scores in human ACLF patients. Spearman correlation coefficients and p-values are indicated. Bars represent mean ± SD; ****P < 0.0001, ***P < 0.001, **P < 0.01, *P < 0. 05, n.s., no significance.

Based on the interconnected metabolic-immune dysfunction patterns, we systematically delineated evolutionarily conserved transcriptional networks driving ACLF progression by constructing cross-species signatures reflective of both pathomechanistic drivers and monocyte/macrophage polarization. To this end, integrative cross-species transcriptomic analysis was performed and prioritized 10 evolutionarily conserved genes exhibiting consistent directional expression shifts across ACLF progression (**Figure 7A**). Among them, six of these genes-*MMP12*, *TREM2*, *ITGA2*, *CAPG*, *F13A1*, and *LGALS3*-are functionally linked to monocyte/macrophage regulation, including migration (*MMP12*, *ITGA2*), phagocytosis (*TREM2*, *LGALS3*), and inflammatory signaling (*CAPG*, *F13A1*), suggesting their collective role in driving myeloid cell activation during ACLF (**Figure 7A**).

STRING-based protein–protein interaction analysis revealed robust functional connectivity among these 10 genes (**Figure 7B**), with GO enrichment further implicating their involvement wound response, leukocyte migration, and immune activation (**Supplementary Figure S9A**). Bulk RNA-seq data confirmed significant upregulation of all six monocyte-associated genes in ACLF across both species (Human: **Supplementary Figure S9B**; Mouse: **Supplementary Figure S9C**), with dynamic temporal patterns: some genes (such as *Capg*, *F13a1*, *Lgals3*, and *Ccr2*) exhibited progressive elevation from fibrosis to ACLF, while *Mmp12* and *Trem2* peaked at intermediate stages (**Supplementary Figure S9D**).

Independent validation by RT-qPCR of six monocyte/macrophage-related genes (*MMP12*, *TREM2*, *ITGA2*, *CAPG*, *F13A1*, and *LGALS3*) and western blot analysis of two markers (*TREM2* and *CCR2*) in clinical and murine cohorts recapitulated these expression trends, demonstrating an increasing trend during ACLF progression (**Figures 7C–E**). External bulk RNA-seq data from a non-HBV ACLF patient validation cohort (GSE139602) confirmed the increasing trend of TREM2 during disease progression (41), consistent with our findings, whereas CCR2 expression showed no significant change (**Supplementary Figure S9E**), indicating that further validation is required when extending these results to ACLF of other etiologies. In a CCl_4_-induced mouse model cohort with multiple time points (GSE222576) (42), *Trem2* and *Ccr2* displayed increased expression during the first 6 weeks followed by a decline thereafter (**Supplementary Figure S9F**). Expression levels of all six genes showed strong positive correlations with MELD scores (**Figure 7F**), underscoring their clinical relevance as ACLF severity biomarkers. These conserved monocyte-centric signatures highlight the key role of myeloid dysregulation in ACLF pathogenesis and identify related therapeutic targets, including *TREM2*-mediated phagocytic reprogramming, *MMP12*-driven matrix remodeling, and the activation and accumulation of disease-associated macrophage and monocyte populations marked by *TREM2* and *CCR2*. The dual utility of these markers, both as prognostic indicators and mechanistic nodes, highlights their translational potential in ACLF management.

## Discussion

Acute-on-chronic liver failure (ACLF) is characterized by rapid hepatic deterioration and high short-term mortality in patients with pre-existing chronic liver disease. However, the molecular mechanisms that drive its onset and progression remain incompletely understood. This study elucidates the intricate interplay between immune dysfunction and metabolic collapse in HBV-associated ACLF through cross-species transcriptomic profiling. The progression of ACLF is marked by a paradoxical immune landscape characterized by simultaneous hyperactivation and exhaustion. Monocyte/macrophage infiltration dominates the hepatic microenvironment, driving sustained proinflammatory signaling through cytokines such as IL-1β and IL-6, while compensatory upregulation of immunosuppressive mediators like IL-10 and TGF-β1 reflects a failed attempt to restore immune equilibrium. This dysfunction likely stems from chronic antigenic stress, where persistent HBV infection perpetuates T cell activation and myeloid cell recruitment, further destabilizing immune homeostasis. The conserved upregulation of antigen-presentation genes across species suggests that adaptive immune responses, though maladaptive, play a central role in exacerbating tissue injury. Such immune exhaustion creates a permissive environment for hepatocellular damage, where unresolved inflammation and impaired regulatory mechanisms converge to accelerate organ failure.

The metabolic perturbations observed in ACLF reveal a systemic breakdown of hepatic function, extending beyond mere energy deficiency. Early suppression of mitochondrial pathways in cirrhosis-particularly those governing oxidative phosphorylation and fatty acid β-oxidation-sets the stage for progressive metabolic paralysis. Disruption of one-carbon metabolism and methyl donor synthesis impairs epigenetic regulation and detoxification, compounding cellular stress. The collapse of gluconeogenesis and steroid biosynthesis further underscores the liver’s inability to maintain systemic metabolic coordination. These defects are not isolated but interact dynamically with immune dysfunction: inflammatory cytokines suppress PPAR signaling, inhibiting lipid metabolism, while metabolic failure limits ATP availability, crippling immune cell effector functions. This bidirectional crosstalk establishes a self-reinforcing cycle, where immune overactivation and metabolic insufficiency mutually exacerbate disease progression.

The identification of evolutionarily conserved gene signatures, particularly monocyte/macrophage-associated markers like MMP12 and TREM2, highlights myeloid cell reprogramming as a pivotal driver of ACLF. These genes, consistently upregulated in both human and murine ACLF, correlate with disease severity and may serve dual roles as biomarkers and therapeutic targets. For instance, TREM2, a regulator of phagocytic function, may promote fibrogenic macrophage polarization, suggesting that targeting such checkpoints could mitigate microenvironmental stress. While murine models replicate terminal-stage metabolic-immune failure with high fidelity, their attenuated early metabolic shifts compared to human cirrhosis likely reflect differences in chronic injury adaptation. Additionally, the murine model exhibits pronounced immune-inflammatory activation superimposed on metabolic perturbations during the ACLF stage, primarily reflecting the early phase of acute decompensation. In contrast, the human ACLF cohort analyzed in this study represents the terminal stage of disease progression, characterized by profound metabolic collapse accompanied by immune exhaustion or suppression. Accordingly, the translational value of the murine model lies in its ability to simulate the prelude to immune-metabolic collapse in ACLF and to provide a platform for evaluating interventions targeting early immune activation and immune - metabolic interactions.

Despite these advances, our study has limitations. Although the murine model differs from human HBV-related etiology, it effectively recapitulates key pathological features of ACLF. The human samples analyzed in this study were limited to patients with HBV-related ACLF. Therefore, whether the observed immune and metabolic alterations are shared by ACLF of other etiologies, such as alcohol- or HCV-associated ACLF, remains unclear and warrants further investigation in future studies. Additionally, bulk transcriptomic profiling cannot distinguish precise cellular subsets or reveal their transcriptional interactions with hepatocytes and liver metabolism. The hepatic microenvironment is highly heterogeneous, and specific myeloid cell states play critical roles in ACLF pathogenesis. This limitation leaves a significant gap in understanding how myeloid immunity interacts with hepatic metabolism during ACLF progression. Therefore, future studies should employ single-cell RNA sequencing or spatial transcriptomics to identify specific pathogenic cell populations in ACLF livers, particularly infiltrating monocyte-derived macrophages, and clarify their metabolic states and contributions to disease progression.

## Conclusion

This work positions ACLF as a syndrome of intertwined immune exhaustion and metabolic bankruptcy, driven by conserved transcriptional networks. Therapeutic strategies must address both axes: restoring mitochondrial resilience to break the energy deficit and recalibrating myeloid cell function to resolve inflammation. By mapping the dynamic interplay of these systems, our findings provide a framework for developing stage-specific interventions, emphasizing the need for combinatorial approaches that target the immune-metabolic nexus. Future research should prioritize translating these insights into therapies that not only mitigate hepatic failure but also restore systemic homeostasis in critically ill ACLF patients.

## Data Availability

The datasets presented in this study are available in the online repository at https://www.ncbi.nlm.nih.gov/geo/ under accession number GSE298435.
