# Supplementary material for: Cross-species hepatic transcriptomics identify conserved immune-metabolic reprogramming in acute-on-chronic liver failure progression
Source: Front Immunol. 2026 Feb 24;17:1702689. doi: 10.3389/fimmu.2026.1702689 (PMC12971639; doi:10.3389/fimmu.2026.1702689)
Supplement: Supplementary file 1 [file DataSheet1.docx]

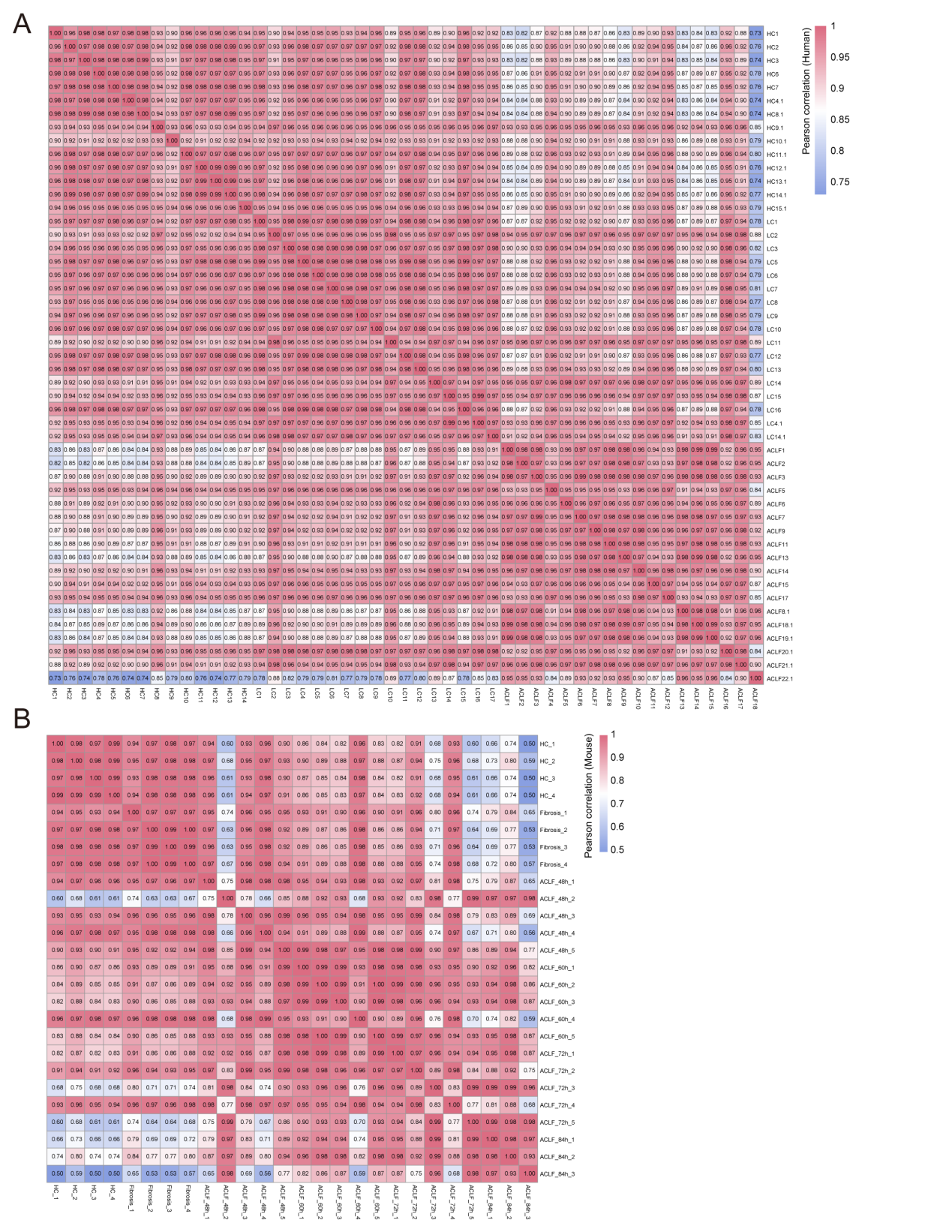


**Figure S1.** Sample-level transcriptomic correlation analysis in human and mouse liver samples. (A) Correlation heatmap of pairwise transcriptomic similarity across all human liver samples (HC, LC, and ACLF). (B) Correlation heatmap showing pairwise similarity among mouse liver samples across HC, Fibrosis, and multiple ACLF timepoints.


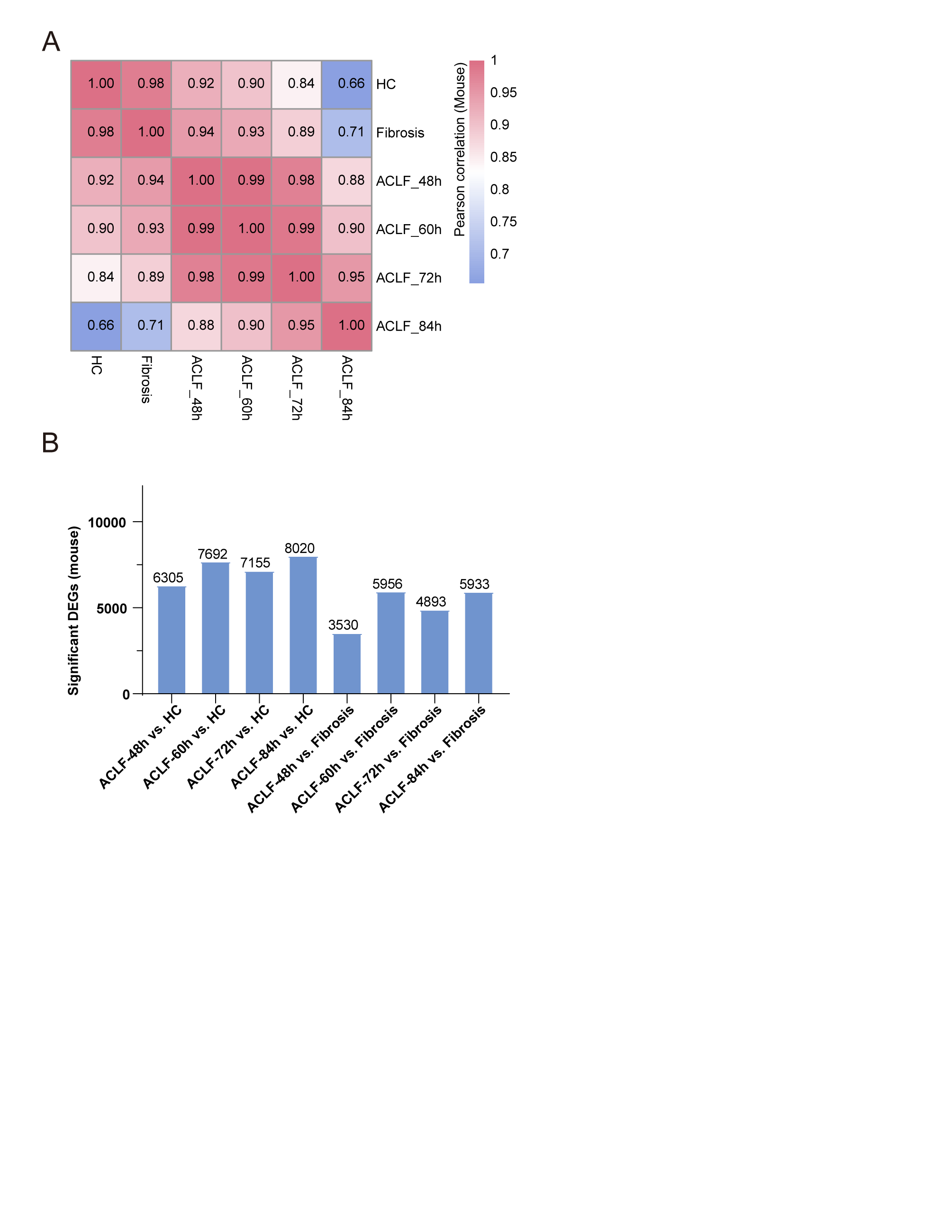


**Figure S2.** Transcriptomic divergence across ACLF timepoints in mouse liver. (A) Correlation heatmap illustrating transcriptomic similarity between mouse group averages, including HC, Fibrosis, and ACLF subgroups (ACLF-48h, ACLF-60h, ACLF-72h, ACLF-84h). (B) Bar plot showing the number of differentially expressed genes (DEGs) in each ACLF subgroup compared to HC and Fibrosis groups.


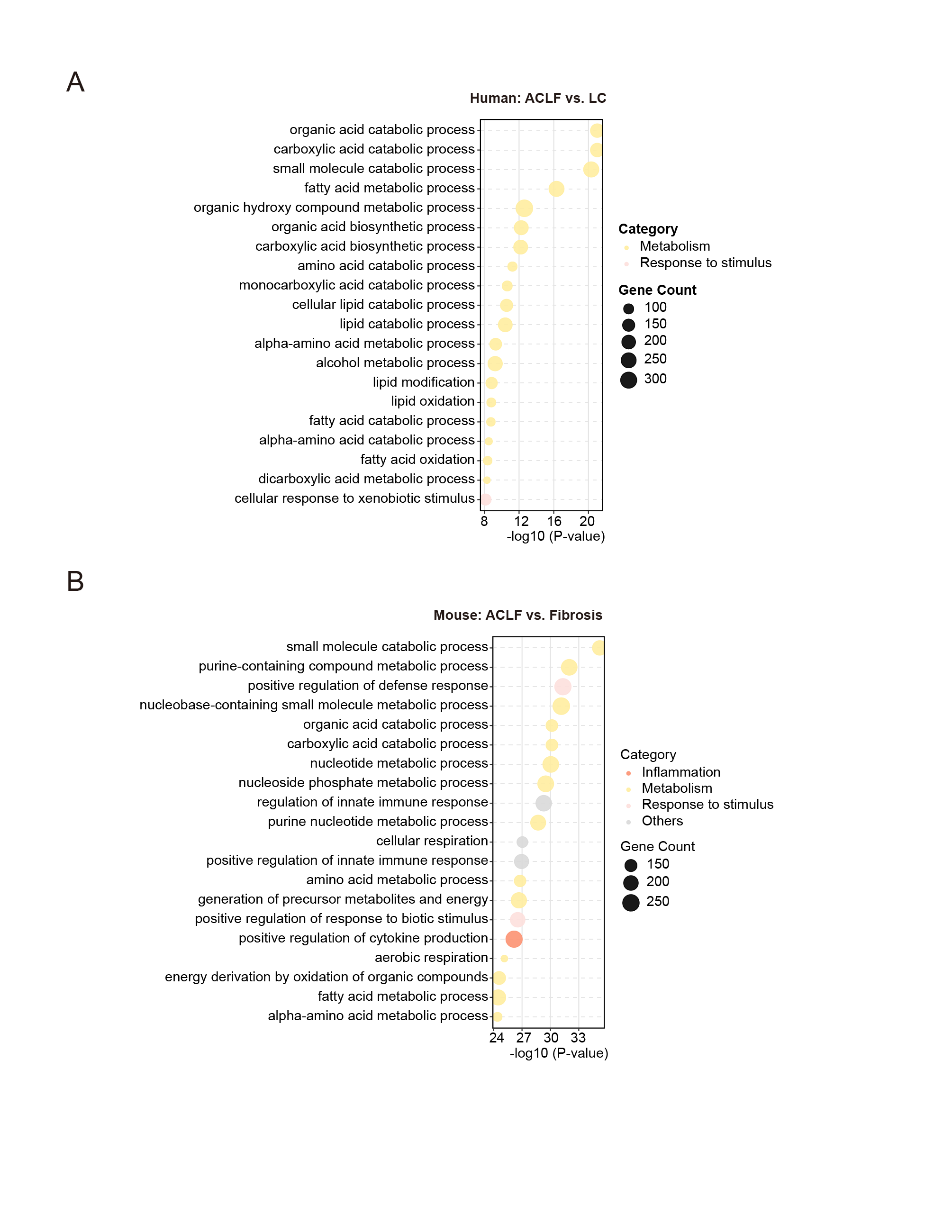


**Figure S3.** Enrichment GO pathway of representative biological processes in human and mouse livers. (A)Top 20 GO-BP terms in the ACLF vs. LC comparison of human liver samples. (B)Top 20 GO-BP terms in the ACLF vs. Fibrosis comparison of mouse liver samples.


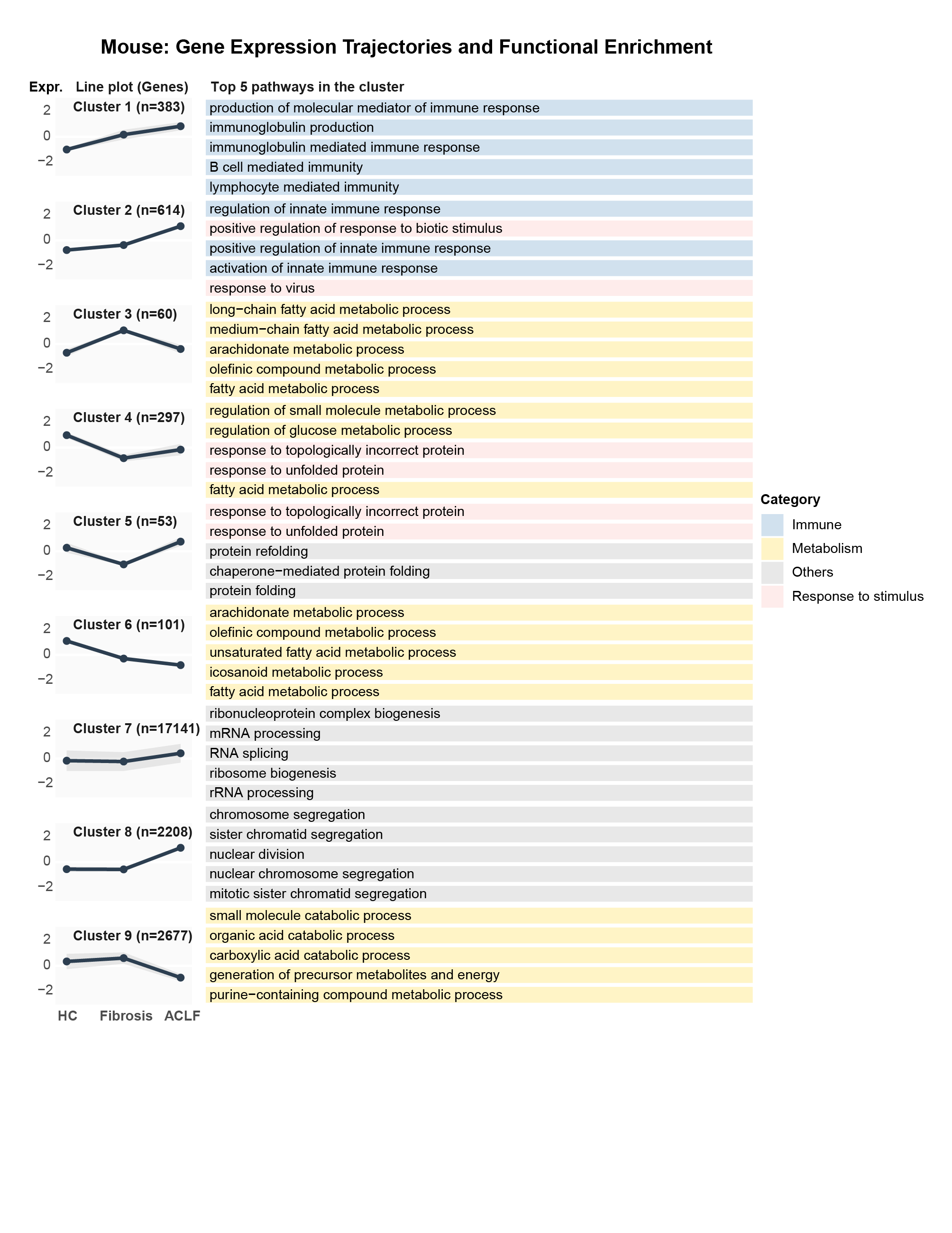


**Figure S4.** Z-score normalized gene expression profiles (9 clusters) and functional enrichment analysis in mice liver samples. Left panel: The solid black line indicates the mean expression level of all genes in the cluster across disease stages (HC, Fibrosis, ACLF), while the shaded ribbon represents the standard deviation. Right panel: Representative top enriched GO biological processes for each cluster. The colored bars indicate the functional category of the GO terms, matching the color scheme in Figure 2.


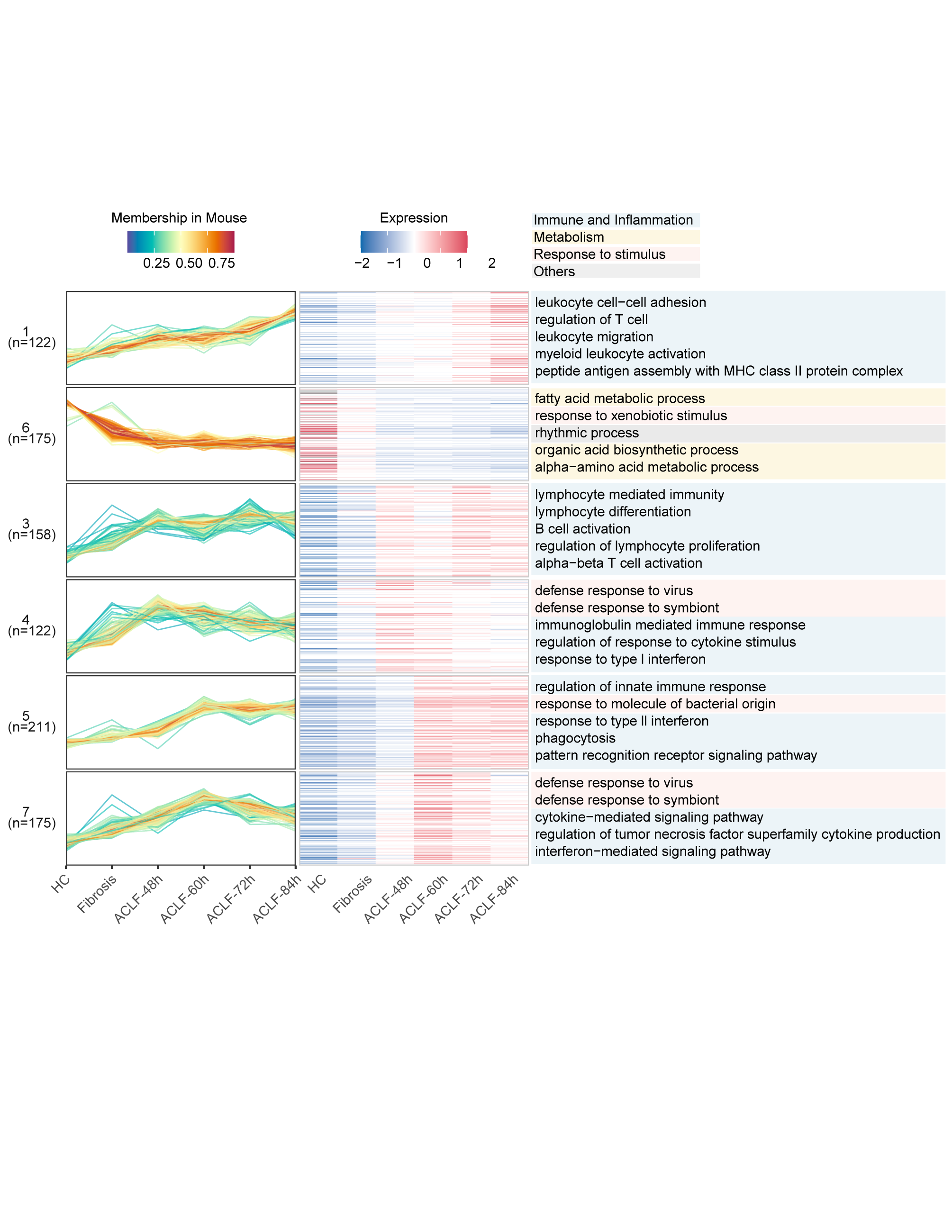


**Figure S5.** Time-series gene expression clustering and functional enrichment in mouse liver transcriptomes.

Time-series clustering of gene expression patterns in mouse liver samples across HC, Fibrosis, and multiple ACLF timepoints (48h, 60h, 72h, 84h) using the Mfuzz algorithm. Each row represents a gene cluster showing temporal expression trends (left panel), mean-centered expression levels (middle heatmap), and representative GO biological processes enriched in each cluster (right panel). Functional categories include immune and inflammation, metabolism, response to stimulus, and other processes.


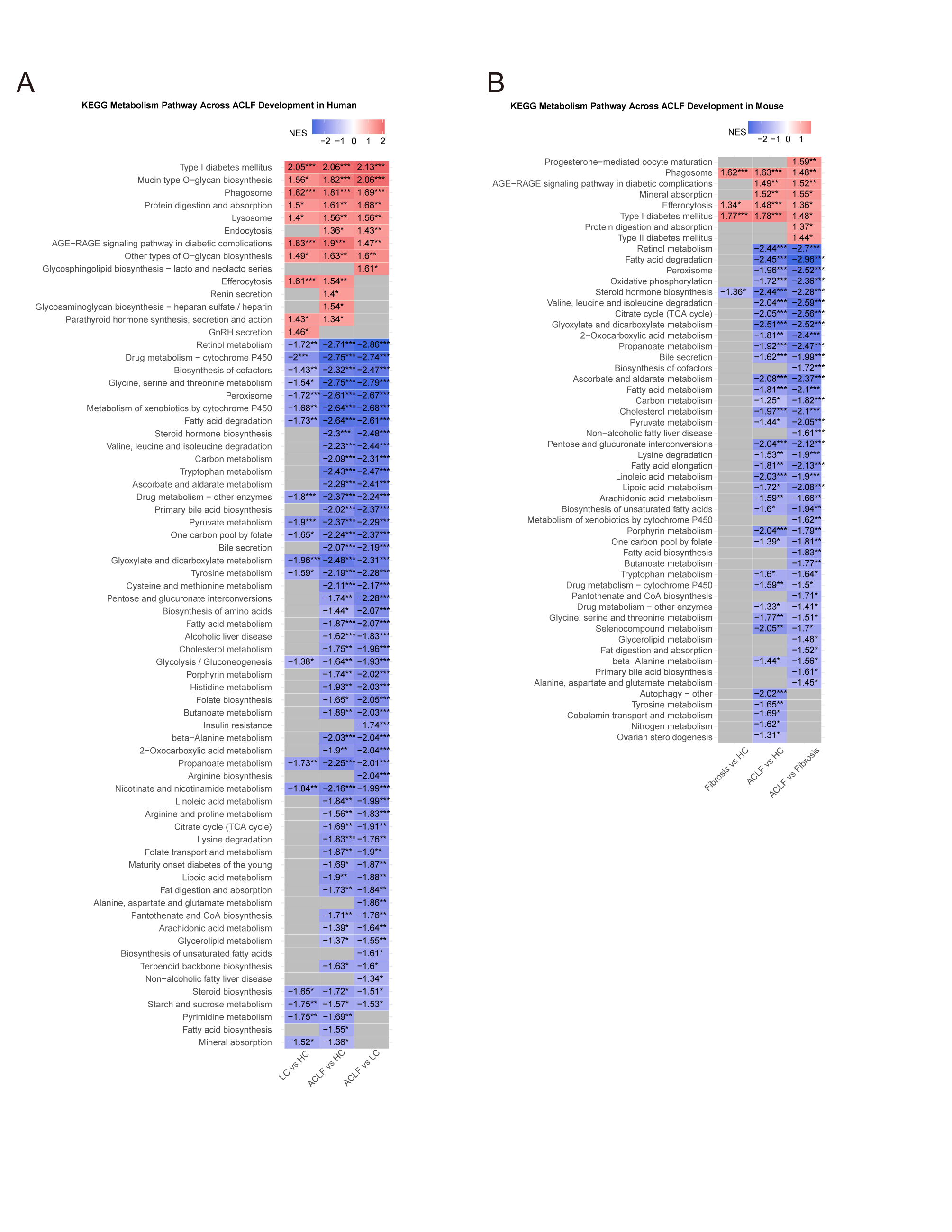
**Figure S6.** Supplementary KEGG pathway analysis of metabolism-related changes during ACLF progression (A) Heatmap of KEGG metabolism-related pathways significantly altered in at least one human liver comparison (LC vs. HC, ACLF vs. HC, or ACLF vs. LC), ranked by normalized enrichment score (NES). Pathways are colored by enrichment direction, with red indicating upregulation and blue indicating downregulation. (B) Heatmap of KEGG metabolism-related pathways significantly altered in at least one mouse liver comparison (Fibrosis vs. HC, ACLF vs. HC, or ACLF vs. Fibrosis), ranked by NES. Pathways are colored by enrichment direction, with red indicating upregulation and blue indicating downregulation.


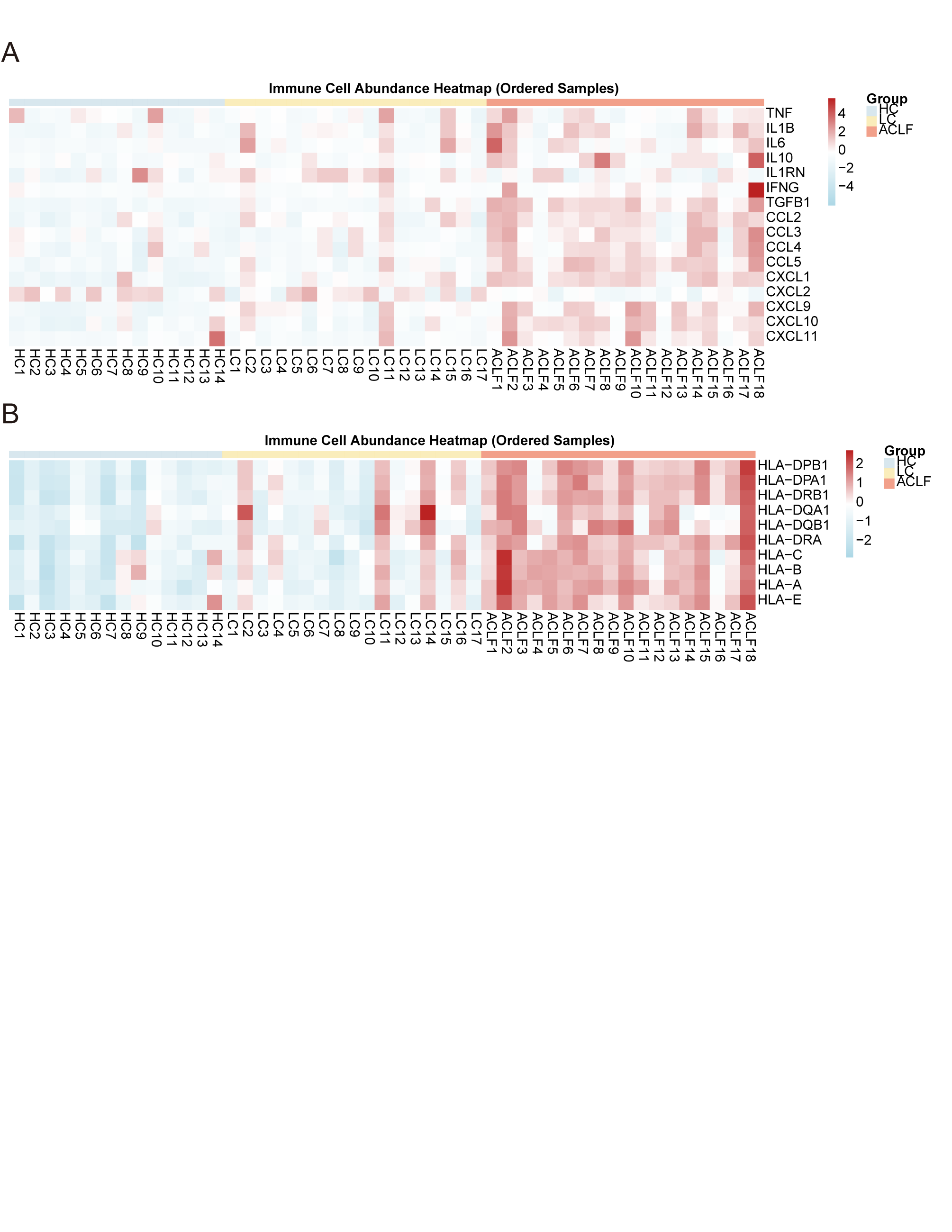


**Figure S7.** Cytokine and antigen presentation gene expression in human livers during ACLF progression

1. Heatmap showing expression patterns of cytokines and chemokines in human liver transcriptomes across HC, LC, and ACLF groups. (B) Heatmap showing expression of MHC class I and class II genes in the same human liver samples.


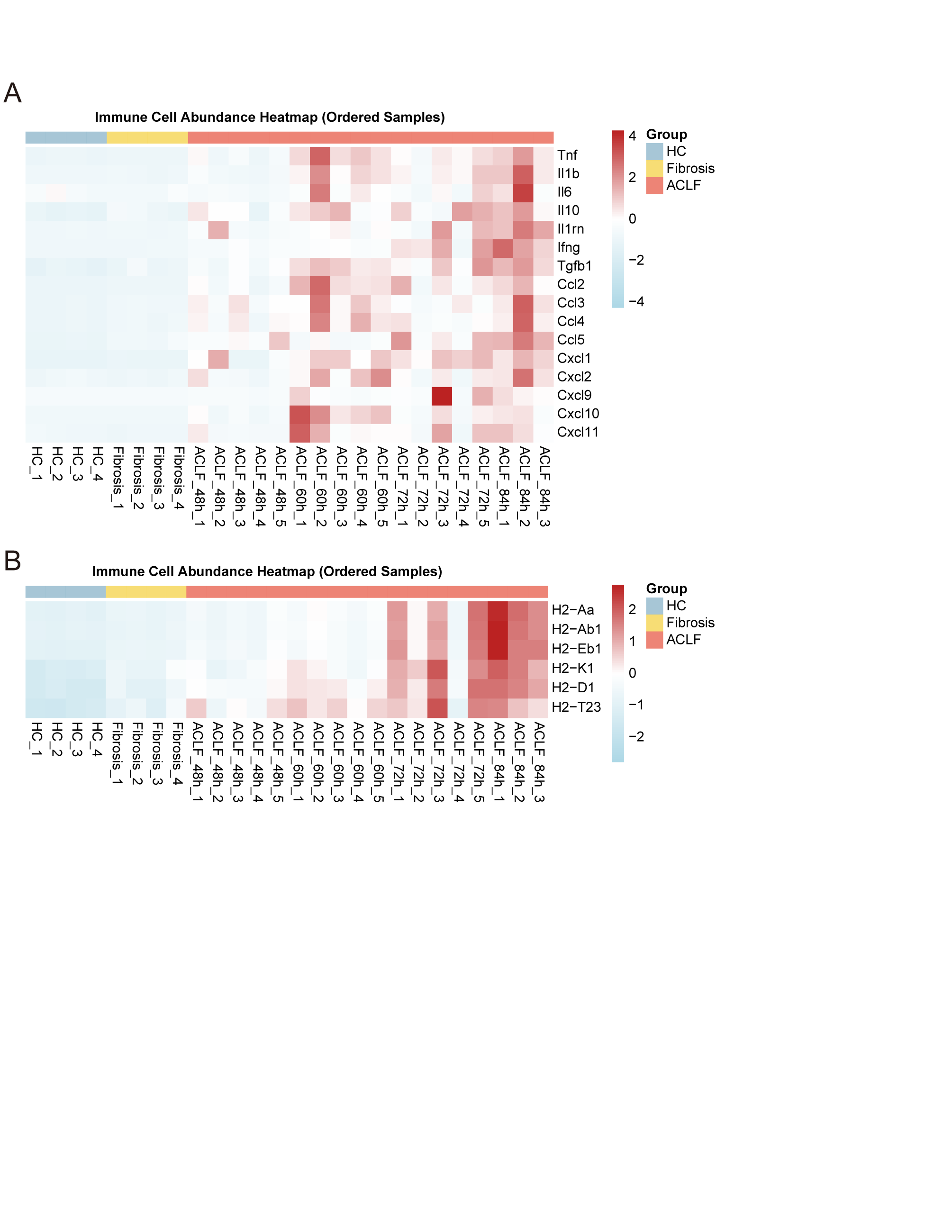


**Figure S8.** Expression heatmaps of immune-related genes in mouse livers. (A) Heatmap showing the expression of cytokines and chemokines across HC, Fibrosis, and ACLF mouse liver samples. (B) Heatmap displaying the expression of both MHC class I and class II genes in mouse livers.


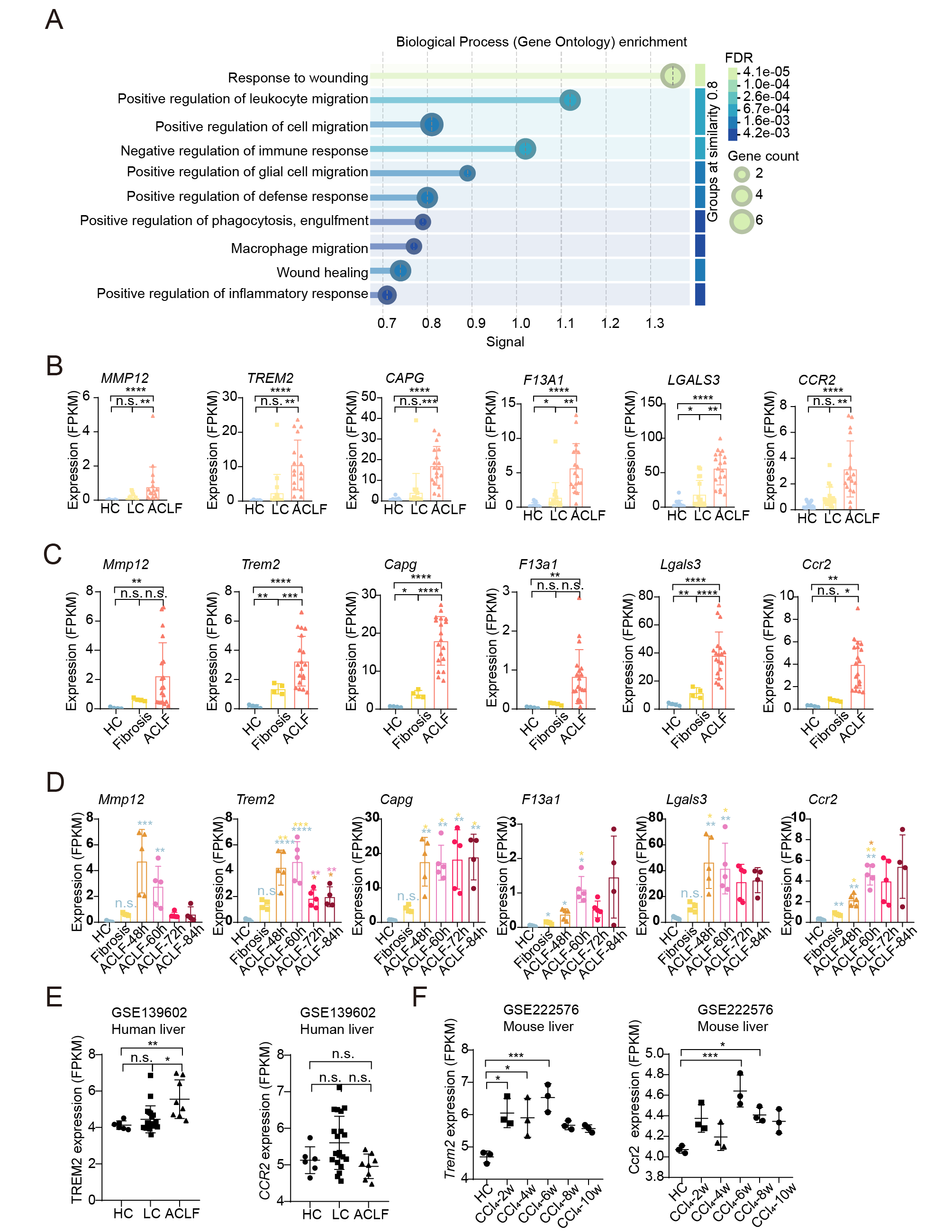


**Figure S9.** Functional relevance and transcriptomic expression patterns of monocyte/macrophage-associated genes. (A) GO biological process enrichment analysis of the top 10 conserved DEGs across human and mouse datasets (as defined in Figure 6). (B) Expression levels (FPKM) of six monocyte/macrophage-related genes (MMP12, TREM2, CAPG, F13A1, LGALS3, CCR2) in human liver transcriptomes across HC, LC, and ACLF groups. (C) Expression levels (FPKM) of the same six genes in mouse liver transcriptomes across HC, Fibrosis, and ACLF groups. (D) Time-resolved expression patterns (FPKM) of the six genes in mouse liver transcriptomes at 48 h, 60 h, 72 h, and 84 h after acute insult during ACLF modeling. (E) Expression level of TREM2 and CCR2 from liver bulk RNA-seq data in non-HBV ACLF patients. (F) Expression level of Trem2 and Ccr2 from liver bulk RNA-seq data in CCl_4_-induced mouse model.
