# Supplementary material for: Cross-species hepatic transcriptomics identify conserved immune-metabolic reprogramming in acute-on-chronic liver failure progression
Source: Front Immunol. 2026 Feb 24;17:1702689. doi: 10.3389/fimmu.2026.1702689 (PMC12971639; doi:10.3389/fimmu.2026.1702689)
Supplement: Supplementary file 2 [file Table1.docx]

**Table S1** Clinical characteristics of patients included in liver RNA sequencing analysis

| Characteristics | | ACLF (n = 18) | LC (n = 17) | HC (n = 14) |
| --- | --- | --- | --- | --- |
| Age (years) | | 49 (39, 48) | 47 (40, 57) | 30 (27, 34) |
| Gender | Male (No.) | 88.9% (16) | 76.5% (13) | 64.3% (9) |
|  | Female (No.) | 11.1% (2) | 23.5% (4) | 35.7% (5) |
|  |  |  |  |  |
| HBV DNA level (IU/ml) | ≤2×10^2^ | 27.8% (5) | 94.1% (16) | - |
|  | 2×10^2^-2×10^6^ | 61.1% (11) | 0.0% (0) | - |
|  | >2×10^6^ | 11.1% (2) | 5.9% (1) | - |
|  |  |  |  |  |
| Laboratory data | Alanine aminotransferase (U/L) | 127.5 (93.5, 281.5) **** | 24.0 (17.5, 52.5) n.s. | 20.0 (15.0, 29.0) |
|  | Aspartate aminotransferase (U/L) | 175.5 (109.0, 357.3) **** | 33.0 (24.0, 61.0) * | 18.5 (15.8, 25.0) |
|  | Albumin (g/L) | 34.6 (31.0, 38.8) **** | 36.1 (31.4, 41.2) **** | 47.4 (44.4, 50.0) |
|  | Total bilirubin (μmol/L) | 525.8 (340.7, 641.2) **** | 23.3 (18.1, 54.5) * | 12.9 (8.7, 14.7) |
|  | Creatinine (μmol/L) | 65.0 (51.0, 74.5) n.s. | 65.0 (51.0, 74.5) n.s. | 62.0 (49.8, 81.3) |
|  | Sodium (mmol/L) | 136.0 (130.0, 141.3) | 143.0 (142.0, 145.5) | - |
|  | White blood cell count (10^9^/L) | 5.9 (3.9, 8.3) n.s. | 3.3 (2.5, 3.9) **** | 6.5 (5.3, 6.9) |
|  | Platelet count (10^9^/L) | 61.5 (42.0, 96.8) **** | 71.0 (38.5, 97.5) **** | 258.5 (215.5, 279.0) |
|  | Haemoglobin (g/L) | 103.5 (96.8, 125.0) **** | 117.0 (86.5, 132.0) ** | 140.0 (129.5, 152.8) |
|  | INR | 2.4 (1.9, 2.6) **** | 1.3 (1.2, 1.5) ** | 0.9 (0.9, 1.0) |
| Severity score (MELD) | | 29 (25, 30) | 12 (10, 15) | - |

Data are presented as the medians (p25, p75) or percentages (numbers of patients).

*****P* < 0.0001, ****P* < 0.001, **P* < 0.05, *n.s.*, no significance for comparisons with the HC group (ACLF or LC vs.HC). INR, international normalized ratio; MELD, Model for End-Stage Liver Disease.

**Table S2** Clinical characteristics of patients included in for the RT-qPCR validation cohort

| Characteristics | | ACLF (n = 5) | LC (n = 5) | HC (n = 5) |
| --- | --- | --- | --- | --- |
| Age (years) | | 47 (36, 54) | 44 (41, 55) | 29 (26, 34) |
| Gender | Male (No.) | 100.00% (5) | 80.00% (4) | 80.00% (4) |
|  | Female (No.) | 0.00% (0) | 20.00% (1) | 20.00% (1) |
|  |  |  |  |  |
| HBV DNA level (IU/ml) | ≤2×10^2^ | 0.00% (0) | 100.00% (5) | - |
|  | 2×10^2^-2×10^6^ | 100.00% (5) | 0.00% (0) | - |
|  | >2×10^6^ | 0.00% (0) | 0.00% (0) | - |
|  |  |  |  |  |
| Laboratory data | Alanine aminotransferase (U/L) | 484.0 (58.0, 1026.0) * | 21.0 (12.5, 29.5) n.s. | 19.0 (17.0, 34.5) |
|  | Aspartate aminotransferase (U/L) | 181.0 (138.0, 234.5) **** | 27.0 (23.0, 37.0) n.s. | 19.0 (18.0, 23.5) |
|  | Albumin (g/L) | 36.1 (29.5, 38.3) ** | 37.3 (34.1, 43.6) * | 45.3 (43.5, 49.8) |
|  | Total bilirubin (μmol/L) | 373.0 (202.8, 537.5**) | 25.2 (15.9, 56.2) n.s. | 8.8 (7.1, 16.7) |
|  | Creatinine (μmol/L) | 58.0 (53.5, 115.0) n.s. | 66.0 (53.0, 103.5) n.s. | 72.0 (48.5, 79.0) |
|  | Sodium (mmol/L) | 138.0 (134.5, 140.0) | 144.0 (141.0, 144.0) | - |
|  | White blood cell count (10^9^/L) | 7.3 (4.4, 7.6) n.s. | 2.44 (1.6, 3.6) n.s. | 6.7 (5.5, 8.1) |
|  | Platelet count (10^9^/L) | 91.0 (43.0, 141.5) n.s. | 42.0 (29.5, 224) n.s. | 203.0 (168.5, 232.0) |
|  | Haemoglobin (g/L) | 124.0 (96.5, 142.0) n.s. | 90.0 (77.5, 213.5) n.s. | 154.0 (142.0, 165.5) |
|  | INR | 2.3 (2.3, 2.8) **** | 1.4 (1.2, 1.5) n.s. | 0.9 (0.9, 1.0) |
| Severity score (MELD) | | 29 (25, 33) | 13.0 (9, 16) | - |

Data are presented as the medians (p25, p75) or percentages (numbers of patients). *****P* < 0.0001, ****P* < 0.001, **P* < 0.05, *n.s.*, no significance for comparisons with the HC group (ACLF or LC vs.HC). INR, international normalized ratio; MELD, Model for End-Stage Liver Disease.
